# GPS or travel diary: Comparing spatial and temporal characteristics of visits to fast food restaurants and supermarkets

**DOI:** 10.1371/journal.pone.0174859

**Published:** 2017-04-07

**Authors:** Jason Y. Scully, Anne Vernez Moudon, Philip M. Hurvitz, Anju Aggarwal, Adam Drewnowski

**Affiliations:** 1 The Urban Form Lab, Department of Urban Design and Planning, University of Washington, Seattle, Washington, United States of America; 2 Center for Public Health Nutrition, Department of Epidemiology, University of Washington, Seattle, Washington, United States of America; Morehouse School of Medicine, UNITED STATES

## Abstract

To assess differences between GPS and self-reported measures of location, we examined visits to fast food restaurants and supermarkets using a spatiotemporal framework. Data came from 446 participants who responded to a survey, filled out travel diaries of places visited, and wore a GPS receiver for seven consecutive days. Provided by Public Health Seattle King County, addresses from food permit data were matched to King County tax assessor parcels in a GIS. A three-step process was used to verify travel-diary reported visits using GPS records: (1) GPS records were temporally matched if their timestamps were within the time window created by the arrival and departure times reported in the travel diary; (2) the temporally matched GPS records were then spatially matched if they were located in a food establishment parcel of the same type reported in the diary; (3) the travel diary visit was then GPS-sensed if the name of food establishment in the parcel matched the one reported in the travel diary. To account for errors in reporting arrival and departure times, GPS records were temporally matched to three time windows: the exact time, +/- 10 minutes, and +/- 30 minutes. One third of the participants reported 273 visits to fast food restaurants; 88% reported 1,102 visits to supermarkets. Of these, 77.3 percent of the fast food and 78.6 percent supermarket visits were GPS-sensed using the +/-10-minute time window. At this time window, the mean travel-diary reported fast food visit duration was 14.5 minutes (SD 20.2), 1.7 minutes longer than the GPS-sensed visit. For supermarkets, the reported visit duration was 23.7 minutes (SD 18.9), 3.4 minutes longer than the GPS-sensed visit. Travel diaries provide reasonably accurate information on the locations and brand names of fast food restaurants and supermarkets participants report visiting.

## Background

Where people purchase the food they eat affects their health. In particular, diet quality and weight status have been linked to the types of restaurants people frequent and the types of stores where they shop [1–3]. Further, higher levels of exposure and/or access to healthy foods have been associated with better health outcomes while the opposite was found for unhealthy foods [4,5]. In this line of research, fast food restaurants (FFRs) are commonly used as examples of unhealthy food places [6] and supermarkets as examples healthy places [7].

People’s exposure/access to the food environment relates to both their activity space (where they live, work and travel) and to the places they self-select to attend to their daily needs [8]. Early work focused on participants’ proximal environments, specifically the food sources in their home neighborhoods. A review of 131 studies on the relationship between the built environment and cardiometabolic outcomes revealed that 90 percent of the studies looked only at exposures near participants’ residences [9].

More recently, GPS and travel data have permitted researchers to explore the potential to patronize food establishments through the framework of exposure/access to food as individuals travel through their daily environments [10–14]. However, these studies often fall short of including the locational and temporal characteristics of exposure to self-selected food places [8]. In such instances, predictors of potential (exposure, access, etc.) will also comprise measurements of the outcome (self-selected visits or the health outcomes related to visits). Thus it is crucial to distinguish between people’s routine exposure/access to the food environment from the food establishments that they willfully select to visit [8].

Surveys have been the traditional instrument to capture self-selected activity related to food shopping or eating out. For example, the American Time Use Survey (ATUS) has found that about 14 percent of the population is ‘usually’ engaged in the grocery shopping (of them 73 percent are women). The average time spent grocery shopping per visit varied from 39 to 43 minutes not including travel. Goodman [15] found that women grocery shopped for an average of 42 minutes compared to 39 minutes for men; and Hamrick et al. [16] found that those under the age of 30 spend 43 minutes compared to those between 30 and 54 who shop for 40 minutes.

Project-specific surveys have included information on the types of food establishments used, the frequency of patronage, and in some cases, the expenses related to the activities [17,18]. Yet, they typically do not provide information on the location and the name of the food places used [19,20]. Used in mobility studies, multiday travel diaries improve on surveys by yielding temporally fine-grained chronological data on activity, as well as more precise definition of places visited (e.g., name of establishment, address, etc.), and mode of travel.

Diaries can be trip-based (measuring the characteristics of the trips respondents take between an origin and a destination); or place-based (measuring the name and address of the places respondents visit as well as their arrival and departure times for each place) [21]. Both types of diaries can also record the activities being performed at a location (e.g., meeting a friend, or eating) and the time spent at or between locations, places, or activities.

Detailed diary data, however, remain self-reported and therefore susceptible to human errors such as recall or social bias. Diaries are now often augmented by GPS—based objective data on time, location, and speed of travel. A few transportation studies have compared diary and GPS data. These studies have examined either the trips people reported taking or the places they reported visiting.

One study of 1,104 travel-diary-reported trips found that only 53.2% of the reported trips had any co-occurring GPS data [22]. The authors posit that inconsistent wear of the GPS devices was responsible the lack of GPS data. Of the trips with GPS data, about 64% had trip origins and destinations that matched those of the diary. Similarly, Chen and colleagues [23] compared travel surveys to GPS records in an attempt to identify the transportation mode of reported trips in New York City. Their success rate ranged from 60 to 95% based on the mode of travel. Kelly et al. [24] aggregated the differences between reported and GPS—measured trip durations from eight studies and found that reported trip durations were 4.4 minutes (28.6%) longer than the GPS trip duration.

Findings from studies of reported places visited had similar results. In a validation study of an activity location questionnaire, researchers found that 75% of self-reported locations were within 400 meters (about a quarter mile) of locations recorded by the GPS data [25]. In another study comparing GPS with reported visits to places researchers found a 100% agreement when the places visited were the participants’ own homes, but they found agreements ranging from 50 to 80% when the place was a commercial or religious establishment, suggesting that recall was place-specific [26]. A third study with a small number of observations comparing parents’ reports of their children’s locations to GPS data yielded a 48% agreement between the two datasets [27].

Though GPS data have been called the “best practical standard” for identifying the location and duration of activity [27], the data are not a gold standard. Participants’ adherence to study protocols cannot be controlled, and data reception is subject to errors (e.g. blocked, interrupted, or redirected communications with satellites which is often related to building architecture) [28]. Further, the locational data from GPS is not descriptive, it is limited to a latitude, longitude, altitude, and speed of travel. The place name of the location either must be inferred from supporting GIS layers or provided by participants in the form of self-report. GPS data also does not include any information about what participants were doing at a specific location.

The present study compared locational data in the form of travel diaries and GPS records from a large urban and suburban population assessed over a seven-day period. Given the importance of accurately measuring exposure and access to healthy and unhealthy food, the locations of interest were fast food restaurants and supermarkets. We used GPS data to verify accuracy of diary entries of individual food establishments visited and the duration of the visits. Based on a novel methodology to match GPS records to reported visits, this study highlights the relative value of detailed self-reported and objectively sensed visits to two establishments shown in previous studies to be related to health [29,30].

## Methods

### Participants

All procedures and protocols used in the study were approved by the University of Washington Institutional Review Board. Both verbal and written consent were obtained from participants. Data came from the Seattle Obesity Study (SOS) II, a longitudinal study examining weight change, the food environment, and mobility patterns in King County, Washington. Parcel-based sampling [31] was used to establish a sampling frame of residential units from the approximately 450,000 parcels within the King County Urban Growth Boundary (UGB). To provide equal distributions by socioeconomic status, residential units were selected based on one of three residential property values (<$199K, >-$200K–$299K, and > = $300K) and on the county-wide ratio of 58 single family to 42 multifamily units. Parcel and assessed property value data came for the King County Assessor [32,33]. A commercial supplier matched addresses to phone numbers. Excluding duplicates and incomplete records, the sampling frame comprised 25,460 addresses and phone numbers.

Potential participants were sent pre-notification postcards followed by up to three telephone calls. Eligible participants were English-speaking, 18 to 55, mobile adults, who were the primary food shoppers in their households. Of the 712 eligible participants, 516 (72.5%) gave their written consent for the study and through an in-person meeting were administered a computer-aided survey and instructed in the use of a GPS receiver and a paper place-based travel diary.

### Food establishment data

Fast food restaurants and supermarkets were identified using Public Health Seattle King County (PHSKC) food permit records for the year 2012. Food permits were geocoded using the King County address point GIS layer (King County GIS Center, 2011) for reference within ArcGIS 10 (ESRI, Redlands, CA, 2010). The permits were classified into establishment types as described elsewhere [34]. Fast food restaurants were nationally and/or regionally recognized chains that lacked table service and sold inexpensive food served in a short time span. Supermarkets were nationally and/or regionally recognized chains that sold a wide range of foods, including canned and frozen foods, fresh produce, and a variety of meat, fish and poultry. Place names were standardized to reflect the brand name of each establishment (e.g., McDonalds, Safeway). The tax parcels on which each of the 573 individual fast food restaurants and 199 supermarkets in the county were located were identified using the 2012 King County tax parcel GIS layer and PostGIS 1.5.3 (The PostGIS Development Group, 2008).

### Travel diary and GPS data

Each record in the travel diary included the name, address, time arrived and time left, and arriving travel mode for each place participants reported visiting during the seven-day assessment period. GPS measures were collected using GPS receivers (Qstarz BT-Q1000XT; Qstarz International Co., Ltd., Taipei, Taiwan) recording longitude, latitude, speed, heading, and satellite information at intervals or 30 seconds or less. Measures were represented in a GIS as points.

In their travel diaries, participants reported 16,433 visits to various places including home, work, recreational facilities, and shopping venues. As with the food permit data, the names of all fast food restaurants and supermarkets in the travel diary were standardized by brand using text matching algorithms. Next, all of the places were aggregated by their unique spellings which resulted in 4,679 distinct place names for all 16,433 reported visits. This list was manually reviewed to identify and correct records with errant or alternative spellings of brand names (e.g. MacDonalds or McD, instead of McDonalds). PostgreSQL 9.19 (The PostgreSQL Global Development Group, 2008) was used to identify travel diary place names corresponding to fast food restaurants and supermarkets in the GIS data.

### Matching analyses

The analyses included participants ≥21 years old, who had complete survey data on personal and household characteristics; >3 days of assessment with both diary and GPS data; and did not work in a fast food restaurant or in a supermarket. To be included in the analyses, reported visits had to be located inside King County, the only location for which we had food establishment data.

A three-step method was used to associate GPS point records with reported visits to food establishments. First, GPS points were *temporally matched* to reported visits by selecting point records with timestamps that were within the reported window created by the arrival and departure times. Second, the temporally matched GPS point records were *spatially matched* in a GIS by identifying the points located inside tax assessor parcels in which food establishments by type were located. Third, reported visits were considered *GPS—sensed* when the food establishment associated with the parcel of the temporally and spatially matched points shared the same brand name as the food establishment reported in the travel diary.

Because participants typically report visit times in multiples of five minutes [21], GPS point records were temporally matched using three time windows: the exact reported time; a +/-10 minute time tolerance (the reported arrival time minus ten minutes and the reported departure time plus 10 minute); and a +/-30 minute time tolerance.

Comparisons relying on chi-square analysis were made between participants who reported one or more visits to food establishments by type during the assessment period and those who did not. The duration of visits was calculated in two ways. The GPS durations were calculated by taking the difference between the timestamps of the first and last GPS point records in each food establishment parcel in every time window. Reported durations came directly from the travel diary and were calculated by subtracting the arrival times from the departure times. Reported duration means and standard deviations were calculated for all reported visits and for the subsets of reported visits that were GPS—sensed in all time windows. Simple Pearson product-moment correlations tested the relationships between reported durations and GPS durations among GPS—sensed visits in all time windows. Differences in mean reported durations for GPS—sensed and unsensed visits were compared using analysis of variance (ANOVA). Simple Pearson product-moment correlations tested the relationships between reported and sensed visit duration, and the parcel size to determine whether participants were simply passing by or through the food place.

## Results

Of the 516 participants in SOSII, the following were excluded from the analytic sample: two were <21 years old; five were working in a supermarket; ten had < three days of assessment; 28 lacked any diary data; six lacked GPS data; five had poor quality data for both travel log and GPS; and 14 had incomplete survey data on personal and household characteristics. After all exclusions, the analytic sample comprised 446 participants.

Among the 446 participants, 150 reported in the travel diary at least one visit to a fast food restaurant, and 393 reported at least one visit to a supermarket (Table 1). Of the sample population, 82.7% was 40 years old or older; 69.3% was female; 79.8% White; 65.2% had a household income <$100,000; 63% had at least a college degree; almost 72% lived in households with two or more adults; the majority (55.8%) was married and did not live with children (53.8%); a slight majority (52%) lived in Seattle while the rest of the sample lived in the smaller cities of King County.

**Table 1:** Sample by reported visits to fast food restaurants and supermarkets.

		Fast Food Restaurants					Supermarkets				
		Participants with = >1 reported visit		Participants with no reported visits			Participants with = >1 reported visit		Participants with no reported visits		
N	Total (%) 446 (100)	n 150	% 100	n 296	% 100	p-value^a^	n 393	% 100	n 53	% 100	p-value^a^
**Age categories**						0.017					0.472
21–39	77 (17.3)	36	24.0%	41	13.9%		71	18.1%	6	11.3%	
40–49	199 (44.6)	66	44.0%	133	44.9%		174	44.3%	25	47.2%	
> = 50	170 (38.1)	48	32.0%	122	41.2%		148	37.7%	22	41.5%	
**Gender**						0.999					0.098
Female	309 (69.3)	104	69.3%	205	69.3%		278	70.7%	31	58.5%	
Male	137 (30.7)	46	30.7%	91	30.7%		115	29.3%	22	41.5%	
**Race**						0.954					0.165
Non-Whites	90 (20.2)	31	20.7%	59	19.9%		75	19.1%	15	28.3%	
Whites	356 (79.8)	119	79.3%	237	80.1%		318	80.9%	38	71.7%	
**Annual household income**						0.401					0.766
<50K	125 (28)	41	27.3%	84	28.4%		108	27.5%	17	32.1%	
50K - <100K	166 (37.2)	62	41.3%	104	35.1%		148	37.7%	18	34.0%	
> = 100K	155 (34.8)	47	31.3%	108	36.5%		137	34.9%	18	34.0%	
**Education**						0.022					0.381
Some college or less	165 (37)	67	44.7%	98	33.1%		142	36.1%	23	43.4%	
College graduates	281 (63)	83	55.3%	198	66.9%		251	63.9%	30	56.6%	
**Adults in household**						0.057					0.659
Lives alone	125 (28)	33	22.0%	92	31.1%		112	28.5%	13	24.5%	
Two or more adults in household	321 (72)	117	78.0%	204	68.9%		281	71.5%	40	75.5%	
**Marital status**						0.118					0.363
Married	249 (55.8)	92	61.3%	157	53.0%		223	56.7%	26	49.1%	
Not married	197 (44.2)	58	38.7%	139	47.0%		170	43.3%	27	50.9%	
**Children in household (age < = 18)**						0.005					0.977
No children	239 (53.6)	66	44.0%	173	58.4%		210	53.4%	29	54.7%	
Children	207 (46.4)	84	56.0%	123	41.6%		183	46.6%	24	45.3%	
**Residential location**						<0.001					0.572
Lives outside Seattle	214 (48)	94	62.7%	120	40.5%		191	48.6%	23	43.4%	
Lives in Seattle	232 (52)	56	37.3%	176	59.5%		202	51.4%	30	56.6%	

^a^ Chi-square analysis.

Chi-square analysis comparing food establishment visitors to nonvisitors identified significant differences (p < 0.05) for fast food restaurant visitation but not for supermarket visitation (Table 1). Those who reported fast food restaurant visits were more likely to be younger (24.0%) than non-visitors (13.9%); to have lower educational attainment (44.7%) compared to non-visitors (33.1%); to be living with children than those who did not report a visit (56.0% versus 41.6%); and to be living outside of Seattle (62.7%).

A total of 273 visits to fast food were reported (Table 2). Using the exact time reported in the travel diary, 178 (65.2%) of the reported visits to fast food restaurants could be temporally and spatially matched to GPS points; and 175 (64.1%) could be GPS-sensed. Using the +/-10-minute and +/- 30-minute tolerances for matching the time recorded in the diary to that of the GPS, 211 (77.3%) and 223 (81.7%) of the fast food visits could be GPS-sensed, respectively. For supermarkets, a total of 1,102 visits were reported (Table 2). Using the exact time reported in the travel diary, 822 (74.6%) of these visits could be temporally and spatially matched with GPS points; and 804 (73%) could be GPS-sensed. Using the +/-10-minute and +/-30-minute tolerances for matching the time window of the diary to that of the GPS, 866 (78.6%) and 885 (80.3%) of the supermarket visits could be sensed by GPS, respectively.

**Table 2:** GPS—sensed visits using travel diary, GPS, and brand names.

	Fast food		Supermarkets	
	Number of visits (%)	Number of participants with ≥ 1 reported visits (%)	Number of visits (%)	Number of participants with ≥ 1 reported visits (%)
**Total number (reported visits and participants)**				
n	273 (100)	150 (100)	1102 (100)	393 (100)
**Visits with temporal-spatial matches**^a^				
No time tolerance	178 (65.2)	112 (74.67)	822 (74.59)	341 (86.77)
+/- 10 min	217 (79.49)	127 (84.67)	894 (81.13)	353 (89.82)
+/- 30 min	231 (84.62)	134 (89.33)	918 (83.3)	357 (90.84)
**GPS—sensed visits**^b^				
No time tolerance	175 (64.1)	109 (72.67)	804 (72.96)	338 (86.01)
+/- 10 min	211 (77.29)^c^	124 (82.67)	866 (78.58)^e^	349 (88.8)
+/- 30 min	223 (81.68)^d^	130 (86.67)	885 (80.31)^f^	354 (90.08)

^(a)^ > one GPS point inside time window and inside a GIS food place parcel.

^(b)^ (a) above + brand name of food establishment in parcel GIS matches that in travel log.

^(c)^ Three visits had two matches each.

^(d)^ Seven visits had two matches each.

^(e)^ One visit had two matches.

^(f)^ Two visits had two matches each.

At the exact time window, 72.7% of participants who reported at least one fast food restaurant visit had one or more GPS—sensed visits (Table 2). The percentages increase to 82.7% and 86.7% at the +/- 10 minute and +/-30 minute tolerances, respectively. For supermarkets, 86%, 88.8%, and 90.1% of the participants who reported at least one visit had at least one GPS-sensed visit, using the exact time, and the +/- 10-minute and +/- 30-minute windows, respectively.

At the exact time window, the mean reported duration of GPS—sensed fast food visits was about 16 minutes (SD 21.6). The GPS-measured mean duration for the same visits was about 3.8 minutes shorter (Table 3). For the +/- 10 minute and +/- 30 minute tolerances the mean reported durations were 1.67 and 1.62 minutes shorter, respectively. At the exact time window, the mean reported duration for GPS—sensed supermarket visits was 24.3 minutes (SD 19), which was 7.4 minutes shorter than the mean GPS duration for the same visit. For the +/- 10 minute and +/-30 minute tolerances, the mean reported durations were 3.37 and 1.95 minutes shorter, respectively.

**Table 3:** Visit durations for GPS—sensed visits (minutes).

	Number of visits	Mean GPS duration (sd)	Mean travel-diary—reported duration (sd)	Pearson’s r (p-value)
**GPS—sensed fast food visits**				
No tolerance	175	12.23 (20.84)	16.06 (21.64)	0.97 (0)
+/- 10 minutes	211	12.8 (20.16)^a^	14.47 (20.21)	0.95 (0)
+/- 30 minutes	223	12.81 (18.22)^a^	14.43 (20)	0.91 (0)
**GPS—sensed supermarket visits**				
No tolerance	804	16.89 (16.39)	24.27 (19)	0.77 (0)
+/- 10 minutes	866	20.3 (17.55) ^a^	23.67 (18.92)	0.76 (0)
+/- 30 minutes	885	21.56 (18.21)^a^	23.51 (18.85)	0.75 (0)

^a^ When a reported visit had multiple matches the GPS durations of matches were averaged.

For GPS—sensed visits, the correlation between the GPS-measured duration and the reported duration of fast food visits ranged from 0.97 (p < 0.001) at the exact time window, to 0.95 (p < 0.001) and 0.91 (p < 0.001) at the +/- 10 minute and +/- 30 minute tolerances (Table 3). For supermarket visits the correlations were smaller; 0.77 (p < 0.001), 0.76 (p < 0.001), and 0.75 (p < 0.001) at the exact time, the +/- 10 minute tolerance, and the +/- 30 minute tolerance, respectively (Table 3).

Considering differences in reported visit durations between GPS—sensed and unsensed visits, unsensed visits to fast food restaurants and supermarkets were significantly shorter than GPS—sensed visits using no time tolerance—6.2 minutes shorter for fast food and 4.3 minutes shorter for supermarkets. Differences were not significant for either fast food or supermarket visits were when using the +/- 10 minute and +/-30 minute time windows (Table 4).

**Table 4:** Reported visit durations in minutes for GPS-sensed and unsensed food establishment visits.

**Reported fast food visits** (n = 273)					
Mean duration all reported visits (sd) = 13.97 (18.72)					
	**Matched reported visits**		**Unmatched reported visits**		
	**Number of visits**	**Mean duration (sd)**	**Number of visits**	**Mean duration (sd)**	**p-value**
**No tolerance**	175	16.06 (21.64)	98	9.84 (9.74)	0.010
**+/- 10 minutes**	211	14.47 (20.21)	62	11.94 (10.82)	0.380
**+/- 30 minutes**	223	14.43 (20)	50	11.44 (8.69)	0.348
**Reported supermarket visits** (n = 1102)					
Mean duration all reported visits (sd) = 23.21 (18.58)					
	**Matched reported visits**		**Unmatched reported visits**		
	**Number of visits**	**Mean duration (sd)**	**Number of visits**	**Mean duration (sd)**	**p-value**
**No tolerance**	804	24.27 (19)	298	19.97 (16.85)	0.001
**+/- 10 minutes**	866	23.67 (18.92)	236	21.25 (16.97)	0.095
**+/- 30 minutes**	885	23.51 (18.85)	217	21.77 (17.22)	0.248

A visual inspection of GPS points identified three primary explanations for why reported fast food visits could not be sensed by GPS (S1 Table). First, GPS points inside parcels with food establishments matching the name reported in the travel diary were outside of the time window reported in the diary. These numbers decreased as the time windows increased, ranging from (varying from 0% to 17.3% of reported fast food visits depending on the time window and 0 to 7.4% for reported supermarket visits). Second was the absence of any GPS points measured during the travel diary time window. About 7.3% of reported fast food visits and 6% of reported supermarket visits lacked any GPS points. Finally, the GPS receiver did not change locations at any period within the time window, indicating that participants were either stationary or did not take the device with them during the reported time of the visit (accounting for 4.4% of fast food visits and 4.9% of supermarket visits in all time windows). In other cases, the visual inspection was unable to determine a reason for why reported visits were not sensed (for all time windows, 3.3% and 1.5% of fast food restaurant visits and supermarket visits, respectively). There were also instances of GPS points that were close to the parcel but not inside it (for all time windows, 1.8% and 6.2% of fast food restaurant visits and supermarket visits, respectively). A very small percentage of reported visits had four additional explanations (accounting for 1.5% and 1.1% of reported fast food restaurant and supermarket visits in all time windows, respectively).

## Discussion

The high proportion of GPS—sensed visits indicated that travel diaries were reasonably accurate in recording the locations and the business names of the fast food and supermarkets visited. The results showed a congruence rate between travel diary and GPS data that was as high, or higher, than those reported in previous studies. In previous studies the rates varied from a low of 48 percent [27] to upwards of 80 percent depending on the location type [26].

For GPS—sensed visits, the reported and GPS durations were significantly correlated, although fast food visits had much higher effect sizes for all time windows. For both food establishment types, the correlations decreased as the time windows increased, and in all cases, the GPS durations were shorter than the reported durations. Similarly, GPS—sensed visits had longer durations than unsensed visits. Although, for both food establishment types these differences were only significant at the exact time window—which also had the largest differences in means between sensed and unsensed visits, again for both food establishment types. Overall, the decreases in mean duration differences from the exact time window to the +/- 10 minute suggest that time tolerances are needed when working with self-reported time measures. At the +/- 10 minute and +/- 30 minute tolerances, the smaller differences between reported and GPS—measured visit durations can be explained by the rounding of reported times and the use of multiples of five in reporting times. Transportation studies also found GPS-measured trip duration to be shorter than reported trip duration [24]. While the difference was larger for trips than for places visited, it suggested that in their reports people inflate both travel and activity durations.

The +/-10 minute time window increased the number of reported visits that could be GPS—sensed by at least 10 percentage points over the measurements done with no time tolerance (to 77.3% for fast foods and 78.2% for supermarkets). In contrast, the +/-30 minute window increased the number of GPS—sensed visits by about 4% for fast food and by less than 1% for supermarket visits, suggesting that this larger tolerance likely exaggerated participants’ error in recording the duration of a visit.

The larger tolerances also increased the possibility that a reported visit might have multiple matches (when GPS points within the time window are located inside two or more parcels both with the same food outlet brand name). At the exact time window, neither fast food nor supermarket visits had multiple matches. There were three fast food visits with multiple matches at the +/- 10 minute tolerance and seven at +/- 30 minutes. For supermarkets there was one visit at +/- 10 minutes and two at +/- 30 minutes. The larger time tolerances captured both actual visits and instances in which people were simply passing through a parcel on their way to somewhere else. Given the small gains from increasing the time tolerance from +/- 10 to +/- 30 minutes along with the increased possibilities for multiple matches, a +/- 10 minute seems to perform the best of the three windows. This finding differed from those of transportation studies where the 30 minute time window yielded better results in matching diary and GPS trip data [22].

Parcel size (fast food median parcel size was 0.8 acres [IQR 0.5–1.2] and 3.4 acre [IQR 1.6–8.9] for supermarkets) was an appropriate spatial unit to capture GPS points related to a visit. Mean GPS travel speeds indicated that within parcels the GPS—sensed visits did not include much travel to and from places: speeds were near mean walking speeds at about 1.3 miles per hour (SD 1.2) for fast food restaurants (S2 Table), and 1.6 mph for supermarkets (SD 1.95) within the +/- 10 min time window (S2 Table). Correlations between parcel size and speed of points were not significant (p > 0.05) for fast food visits (S3 Table), and although they were significant for supermarket visits at the exact time window and +/- 10 minutes tolerance (S3 Table), the correlations were small (in both cases r = -0.08). Correlations between parcel size and visit durations showed a similar pattern but with higher effect sizes (S3 Table). For supermarket visits at the +/- 10 minute tolerance the correlation was 0.22 (p < 0.001) for reported duration and 0.34 (p < 0.001) for GPS-measured duration (S3 Table). People spend more time on larger supermarket parcels than they do on smaller ones. This is perhaps related to the higher speeds of travel on smaller supermarket parcels.

Our supermarket shopping visit duration findings were surprisingly different from those of ATUS, in which reported time spent grocery shopping was more than twice as long as either our reported or GPS-sensed visits. In ATUS, time spent in activity (including grocery shopping) excludes traveling to and from the activity. The difference suggested that in ATUS, people considered grocery shopping as a discrete activity, which was not associated with all visits to supermarkets, because the latter might include picking up a take-away meal, odds and ends for a special meals, or shopping for household items.

The study was limited to visits that were reported in travel diaries and therefore might suffer from recall or social bias, the latter bias being more likely for fast food restaurant (recognized as unhealthy places) than supermarket (healthy places) visits. Future studies should examine GPS traces to find out whether participants spent time in or near food establishments during the assessment period, which could help identify possible unreported visits. Using GPS data to identify willful visits to food establishments without the aid of self-reported data is tricky. While GPS points inside a parcel associated with a food establishment may indicate a visit, if there are two or more food establishments on the parcel it may be impossible to determine which food establishment was visited. Longer durations spent inside a parcel may also indicate a visit, however given that time spent in a parcel is estimated by the number of GPS points, longer visits will be more reliably measured than shorter visits. The average length of a visit to a fast food restaurant drive through window is 189.5 seconds [35]. With 30-second intervals such a visit could be represented by six GPS points or fewer.

Furthermore, both diary and GPS data are limited in their ability to characterize habitual behavior. The sample visited fast food restaurants an average of 0.34 (SD 0.47) times a week, among the 150 participants who reported visits the average was 1.8 (SD 1.3) visits a week. For supermarket visits the sample average was 0.88 (SD 0.32) visits per week and among those who reported one or more supermarket visits it was 2.8 (SD 1.8). In comparison, the Food Marketing Institute estimated that consumers average 1.6 supermarket visits per day [36]. No such data exist for fast food restaurant patronage, although eating at fast food restaurants two or more times a week has been shown to affect health [37], and increases in weekly consumption of fast food were positively associated with BMI in young adults [38].

Another limitation of this and similar studies is the possibility that carrying the GPS receiver incentivizes participants to be more accurate in reporting their travel diary entries. The accuracy of self-reported locational data without accompanying GPS data may therefore be lower than it would be with GPS data. This limitation speaks to the complimentary nature of these two data sources, each with their distinctive strengths and weaknesses. While GPS data offer objective locational and travel information, self-reported data can offer descriptive information about locations visited, mode of travel, and what participants were doing at said locations. When used in tandem GPS and self-report are useful tools in controlling for selective daily mobility in exposure studies [8].

## Conclusion

More than 77% of visits to fast food restaurants and supermarkets that were reported in travel diaries could be verified by GPS and GIS in terms of their location and individual establishments being patronized. GPS—sensed visit durations were only 11.5% and 14.2% shorter than those reported for fast food restaurants and supermarkets, respectively. This suggested that travel diaries were a reasonable instrument to capture exposure by self-selection to the two types of places. However, it is important to remember that participants in our study may have been more accurate in filling out their travel diaries because they were also wearing GPS receivers during the observation period.

While travel diaries are more burdensome on participants, and suffer from recall and social bias, they are more cost effective to administer than GPS. They also offer a major advantage over GPS data in that they can be used to measure the activities performed at certain locations, not just the locational information. This is crucial in the study of exposure and access to food establishments as it is necessary to separate willful visits from mere exposure or access (i.e. simply being in the proximity of food establishments). Toward this end, travel diary and GPS data should be treated as complimentary with diaries providing descriptive information about locations visited and the activities performed there and GPS data providing a less biased measure of all the places that participants had the option to visit but did not.

## Supporting information

S1 Table Explanations for reported visits that were not GPS—sensed. (DOCX)

S2 Table Descriptive statistics of GPS—sensed visits to fast food restaurants and supermarkets. (DOCX)

S3 Table Correlations among GPS-sensed variables of matched visits to fast food restaurants and supermarkets. (DOCX)

S1 Travel Diary Seattle Obesity Study II seven-day travel log. (PDF)
