# Supplementary material for: GPS or travel diary: Comparing spatial and temporal characteristics of visits to fast food restaurants and supermarkets
Source: PLoS One. 2017 Apr 7;12(4):e0174859. doi: 10.1371/journal.pone.0174859 (PMC5384745; doi:10.1371/journal.pone.0174859)
Supplement: S1 Table — (DOCX) [file pone.0174859.s001.docx]

**S1 Table. Explanations for reported visits that were not GPS–sensed**

|  | **Number of reported visits (% of reported visits)** | | | | | |
| --- | --- | --- | --- | --- | --- | --- |
|  | **No tolerance** | | **+/- 10 minutes** | | **+/- 30 minutes** | |
|  | **Fast food** | **Supermarkets** | **Fast food** | **Supermarkets** | **Fast food** | **Supermarkets** |
| **Number of reported visits** | **273 (100)** | **1102 (100)** | **273 (100)** | **1102 (100)** | **273 (100)** | **1102 (100)** |
| **Explanation** |  |  |  |  |  |  |
| Points inside parcel were outside reported time window | 48 (17.6) | 81 (7.4) | 12 (4.4) | 19 (1.7) | 0 (0.0) | 0 (0.0) |
| No GPS data | 20 (7.3) | 66 (6.0) | 20 (7.3) | 66 (6.0) | 20 (7.3) | 66 (6.0) |
| GPS receiver never moved | 12 (4.4) | 54 (4.9) | 12 (4.4) | 54 (4.9) | 12 (4.4) | 54 (4.9) |
| Unable to determine | 9 (3.3) | 17 (1.5) | 9 (3.3) | 17 (1.5) | 9 (3.3) | 17 (1.5) |
| Points close to but not inside parcel | 5 (1.8) | 68 (6.2) | 5 (1.8) | 68 (6.2) | 5 (1.8) | 68 (6.2) |
| Reported food place name and food permit name differ but both are appropriate | 3 (1.1) | 1 (0.1) | 3 (1.1) | 1 (0.1) | 3 (1.1) | 1 (0.1) |
| No parcel record | 1 (0.4) | 3 (0.3) | 1 (0.4) | 3 (0.3) | 1 (0.4) | 3 (0.3) |
| GPS receiver error | 0 (0.0) | 2 (0.2) | 0 (0.0) | 2 (0.2) | 0 (0.0) | 2 (0.2) |
| Went to a different food place than the one named | 0 (0.0) | 6 (0.5) | 0 (0.0) | 6 (0.5) | 0 (0.0) | 6 (0.5) |
| **Total reported visits not GPS–sensed** | **98 (35.9)** | **298 (27.0)** | **62 (22.7)** | **236 (21.4)** | **50 (18.3)** | **217 (19.7)** |
