# Supplementary material for: GPS or travel diary: Comparing spatial and temporal characteristics of visits to fast food restaurants and supermarkets
Source: PLoS One. 2017 Apr 7;12(4):e0174859. doi: 10.1371/journal.pone.0174859 (PMC5384745; doi:10.1371/journal.pone.0174859)
Supplement: S2 Table — (DOCX) [file pone.0174859.s002.docx]

**S2 Table. Descriptive statistics of GPS–sensed visits to fast food restaurants and supermarkets^a^**

|  | **Fast food** | | | | |
| --- | --- | --- | --- | --- | --- |
| **Variable** | **Mean** | **SD** | **Median** | **IQR** | **Max** |
| **No tolerance** |  |  |  |  |  |
| GPS duration (minutes) | 12.2 | 20.84 | 4.67 | 2.08–13.8 | 156 |
| GPS speed (mph) | 1.04 | 1.1 | 0.72 | 0.34–1.34 | 7.16 |
| # of GPS points | 55.8 | 120.32 | 19 | 10–39 | 940 |
| Parcel area (sq ft) | 108000 | 223507.1 | 36400 | 23900–51500 | 1560000 |
| **+/- 10 minutes** |  |  |  |  |  |
| GPS duration (minutes) | 12.8 | 20.16 | 6 | 3.16–13.8 | 160 |
| GPS speed (mph) | 1.31 | 1.16 | 0.99 | 0.535–1.68 | 6.86 |
| # of GPS points | 56.6 | 115.73 | 24 | 12–44 | 960 |
| Parcel area (sq ft) | 101000 | 211396.8 | 35000 | 22600–52400 | 1560000 |
| **+/- 30 minutes** |  |  |  |  |  |
| GPS duration (minutes) | 12.8 | 18.22 | 6.38 | 3.5–14.2 | 160 |
| GPS speed (mph) | 1.31 | 1.18 | 1.01 | 0.525–1.71 | 6.86 |
| # of GPS points | 53.8 | 102.07 | 25 | 14–44 | 950 |
| Parcel area (sq ft) | 101000 | 211990.9 | 33900 | 22100–52100 | 1560000 |
|  | **Supermarkets** | | | | |
| **Variable** | **Mean** | **SD** | **Median** | **IQR** | **Max** |
| **No tolerance** |  |  |  |  |  |
| GPS duration (minutes) | 16.9 | 16.39 | 12.2 | 5.36–24.3 | 145 |
| GPS speed (mph) | 1.57 | 1.95 | 1.15 | 0.68–1.83 | 32.3 |
| # of GPS points | 65.1 | 78.08 | 38 | 15–88 | 649 |
| Parcel area (sq ft) | 255000 | 239695.4 | 148000 | 75500–425000 | 1260000 |
| **+/- 10 minutes** |  |  |  |  |  |
| GPS duration (minutes) | 20.3 | 17.55 | 16.3 | 8.18–28.1 | 145 |
| GPS speed (mph) | 1.71 | 1.69 | 1.31 | 0.82–2.06 | 20.6 |
| # of GPS points | 74 | 83.59 | 45 | 19–100 | 656 |
| Parcel area (sq ft) | 247000 | 237759.2 | 146000 | 71300–386000 | 1260000 |
| **+/- 30 minutes** |  |  |  |  |  |
| GPS duration (minutes) | 21.6 | 18.21 | 17 | 8.67–30.4 | 145 |
| GPS speed (mph) | 1.71 | 1.72 | 1.31 | 0.82–2.05 | 20.6 |
| # of GPS points | 77.3 | 87.38 | 47 | 20–101 | 659 |
| Parcel area (sq ft) | 246000 | 237269.1 | 146000 | 71300–386000 | 1260000 |

^a^ When a reported visit had multiple matches the GPS durations of matches were averaged.
