# Supplementary material for: GPS or travel diary: Comparing spatial and temporal characteristics of visits to fast food restaurants and supermarkets
Source: PLoS One. 2017 Apr 7;12(4):e0174859. doi: 10.1371/journal.pone.0174859 (PMC5384745; doi:10.1371/journal.pone.0174859)
Supplement: S3 Table — (DOCX) [file pone.0174859.s003.docx]

**S3 Table. Correlations among GPS-sensed variables of matched visits to fast food restaurants and supermarkets^a^**

|  | **Pearson’s r (p-value)** | | | | | |
| --- | --- | --- | --- | --- | --- | --- |
|  | **Fast food** | | | **Supermarkets** | | |
| **Comparison** | **No tolerance** | **+/- 10 min** | **+/- 30 min** | **No tolerance** | **+/- 10 min** | **+/- 30 min** |
| Speed and reported duration | -0.25 (0.001) | -0.29 (< 0.001 | -0.28 (< 0.001) | -0.09 (0.009) | -0.09 (0.008) | -0.09 (0.008) |
| Speed and GPS duration | -0.32 (< 0.001) | -0.37 (< 0.001) | -0.32 (< 0.001) | -0.22 (< 0.001) | -0.25 (< 0.001) | -0.23 (< 0.001) |
| Speed and duration difference | 0.26 (0.001) | 0.23 (0.001) | 0.03 (0.627) | 0.15 (< 0.001) | 0.21 (< 0.001) | 0.19 (< 0.001) |
| Speed and parcel size | 0.07 (0.334) | 0.08 (0.262) | 0.13 (0.048) | -0.08 (0.025) | -0.08 (0.026) | -0.06 (0.061) |
| Parcel size and reported duration | 0.03 (0.732) | 0.04 (0.566) | 0.03 (0.681) | 0.22 (< 0.001) | 0.22 (< 0.001) | 0.22 (< 0.001) |
| Parcel size and GPS duration | 0.04 (0.569) | 0.09 (0.184) | 0.13 (0.06) | 0.3 (< 0.001) | 0.34 (< 0.001) | 0.34 (< 0.001) |
| Parcel size and duration difference (Reported – sensed duration) | -0.06 (0.405) | -0.17 (0.013) | -0.21 (0.002) | -0.06 (0.092) | -0.15 (< 0.001) | -0.16 (< 0.001) |

^a^ When a reported visit had multiple matches the GPS durations of matches were averaged.
