# Supplementary material for: GPS or travel diary: Comparing spatial and temporal characteristics of visits to fast food restaurants and supermarkets
Source: PLoS One. 2017 Apr 7;12(4):e0174859. doi: 10.1371/journal.pone.0174859 (PMC5384745; doi:10.1371/journal.pone.0174859)
Supplement: S1 Travel Diary — (PDF) [file pone.0174859.s004.pdf]

Thank you for your participation!

SOS II

TRAVEL LOG

Please use for 7 days when wearing the GPS.

ID #

____

Planned Start Day:	Planned End Day:
______________	______________

Travel Log Instructions

See the example of a completed Log on the following pages.

Use this Log to record **PLACES** visited, **HOW YOU GOT THERE**, and the **FOOD RELATED ACTIVITIES** you did at each place. For each place you go, ask yourself, “Where did I go? What was I doing there? How did I get there? What time did I arrive? What time did I leave?”

Record the following in as much detail as possible:

- Places: A place is **any location you travel to**, even if it’s for only a few minutes. It is very important to record place name, address and/or cross-streets and city. If you know the zip code, please record that also. Places can include bus stops, train stations, or park-and-ride facilities (i.e., places where you change travel modes).
- Times: Record the time you arrived at and left each place. Exact times (to the minute) are preferred. For travel log purposes, **each day begins at 3am.**
- Food Related Activities: Record what you are doing at each place you go to using the **“What did I Do”** check box**.** You can record up to 3 activities.
- Modes: Record how (e.g. car, bus) you traveled to each place using the **“How I Got Here”** check boxes.

Starting the Travel Log:

Step 1: Enter the name and address of common places on the next page (home, work, usual bus stops, etc). When you fill out the daily log, you can enter the name of these common places without re-writing the address each time.

Step 2: Review the “How I Got Here” and “What Did I Do” check boxes in the example on pages 1-6. Please check the travel and activity for each place.

Daily Entries:

- Write in the date and check day of the week.
- Write in the time you put on your GPS**!!!
- The first entry is where you started your day (usually home)
- For each place you go,
    - Check a Common Place *or* write in place name and address.
    - Check an activity = what you did. If you are doing more than one activity, check all activities. Limit to 3 activities at each place.
    - Write in the travel mode = how you got there
    - Enter the time arrived and time left.
- At the end of each day, write in the time you took the GPS off.

**The GPS *must* be charged every night, after you take it off for the day. Refer to your GPS instructions or give us a call if you have any questions.

We guarantee that the information you provide will be kept confidential. Information you provide will be labeled using your ID#. Your name will not be connected to the information you provide.

Common Places

- Enter the address of your home, work (if applicable), and school (if applicable), and any other places you visit **frequently**.
- When completing the travel log, you can refer to these places by name. You do not need to write the address again in the log if it is listed below.

Location	Name	Address (i.e. 3124 59th Ave NE, Kent, WA 98030)
Home:		
Work:		
School:		
Other:		

Location	Name	Address (i.e. 3124 59th Ave NE, Kent, WA 98030)
Other:		
Other:		
Other:		
Other:		
Other:		
Other:		
Other:		
Other:		
Other:		
Other:		

EXAMPLE (PLEASE DO NOT FILL OUT)

Date (m/d/y) 9/22/11

Time you put the GPS on: 8:15 AM / PM (AM circled)

**Start of Day:**	**Place Name:** Home	
☒ Home<br>☐ Other:<br>☐ Work<br>☐ School	Full address or two nearest cross streets<br>City: Zip:	
**What did I do:**<br>☒ Snack ☐ Meal ☐ Beverage ☐ Food Shopping		**Time Left:**<br>8:50 AM / PM (AM circled)

**Place #1:**	**Place Name:** Work	**Time Arrived:**<br>10:00 AM / PM (AM circled)
☐ Home<br>☐ Other:<br>☒ Work<br>☐ School	Full address or two nearest cross streets<br>City: Zip:	
**How I got here:**<br>☐ Car ☐ Bus/Transit ☒ Bicycle ☐ Walking ☐ Other________		
**What did I do:**<br>☒ Snack ☒ Meal ☐ Beverage ☐ Food Shopping		**Time Left:**<br>6:00 AM / PM (PM circled)

EXAMPLE (PLEASE DO NOT FILL OUT)

**Place #2:**	**Place Name:** Trader Joes	**Time Arrived:**<br>6:18 AM / PM (PM circled)
☐ Home<br>☒ Other:<br>☐ Work<br>☐ School	Full address or two nearest cross streets<br>45th St and Roosevelt Ave<br>City: Seattle Zip: 98105	
**How I got here:**<br>☐ Car ☐ Bus/Transit ☒ Bicycle ☐ Walking ☐ Other________		
**What did I do:**<br>☒ Snack ☐ Meal ☐ Beverage ☒ Food Shopping		**Time Left:**<br>6:40 AM / PM (PM circled)
**Place #3:**	**Place Name:** Home	**Time Arrived:**<br>7:55 AM / PM (PM circled)
☒ Home<br>☐ Other:<br>☐ Work<br>☐ School	Full address or two nearest cross streets<br>City: Zip:	
**How I got here:**<br>☐ Car ☐ Bus/Transit ☒ Bicycle ☐ Walking ☐ Other________		
**What did I do:**<br>☐ Snack ☒ Meal ☒ Beverage ☐ Food Shopping		**Time Left:**<br>9:00 AM / PM (PM circled)

 EXAMPLE (PLEASE DO NOT FILL OUT)

**Place #4:**<br>☐ Home<br>☒ Other:<br>☐ Work<br>☐ School	**Place Name:** AMC Theatre<br>Full address or two nearest cross streets<br>Pine Street and 6th Ave<br>City: Seattle Zip: 98101	**Time Arrived:**<br>9:20 AM / (PM)
**How I got here:**<br>☒ Car ☐ Bus/Transit ☐ Bicycle ☐ Walking ☐ Other________		
**What did I do:**<br>☒ Snack ☐ Meal ☐ Beverage ☐ Food Shopping		**Time Left:**<br>11:30 AM / (PM)
**Place #5:**<br>☒ Home<br>☐ Other:<br>☐ Work<br>☐ School	**Place Name:** Home<br>Full address or two nearest cross streets<br>City: Zip:	**Time Arrived:**<br>11:45 AM / (PM)
**How I got here:**<br>☒ Car ☐ Bus/Transit ☐ Bicycle ☐ Walking ☐ Other________		
**What did I do:**<br>☐ Snack ☐ Meal ☐ Beverage ☐ Food Shopping		**Time Left:**<br>________AM / PM

EXAMPLE (PLEASE DO NOT FILL OUT)

**Place #6:**<br>☐ Home<br>☐ Other:<br>☐ Work<br>☐ School	**Place Name:**<br>Full address or two nearest cross streets<br>City: Zip:	**Time Arrived:**<br>________ AM / PM
**How I got here:**<br>☐ Car ☐ Bus/Transit ☐ Bicycle ☐ Walking ☐ Other________		
**What did I do:**<br>☐ Snack ☐ Meal ☐ Beverage ☐ Food Shopping		**Time Left:**<br>______AM / PM
**Place #7:**<br>☐ Home<br>☐ Other:<br>☐ Work<br>☐ School	**Place Name:**<br>Full address or two nearest cross streets<br>City: Zip:	**Time Arrived:**<br>______ AM / PM
**How I got here:**<br>☐ Car ☐ Bus/Transit ☐ Bicycle ☐ Walking ☐ Other________		
**What did I do:**<br>☐ Snack ☐ Meal ☐ Beverage ☐ Food Shopping		**Time Left:**<br>________AM / PM

 EXAMPLE (PLEASE DO NOT FILL OUT)

**Place #8:**<br>☐ Home<br>☐ Other:<br>☐ Work<br>☐ School	**Place Name:**<br><br>Full address or two nearest cross streets<br><br>City: Zip:	**Time Arrived:**<br>__________AM / PM
**How I got here:**<br>☐ Car ☐ Bus/Transit ☐ Bicycle ☐ Walking ☐ Other____		
**What did I do:**<br>☐ Snack ☐ Meal ☐ Beverage ☐ Food Shopping		**Time Left:**<br>__________AM / PM
**Place #9:**<br>☐ Home<br>☐ Other:<br>☐ Work<br>☐ School	**Place Name:**<br><br>Full address or two nearest cross streets<br><br>City: Zip:	**Time Arrived:**<br>_______ AM / PM
**How I got here:**<br>☐ Car ☐ Bus/Transit ☐ Bicycle ☐ Walking ☐ Other__________________		
**What did I do:**<br>☐ Snack ☐ Meal ☐ Beverage ☐ Food Shopping		**Time Left:**<br>__________AM / PM

EXAMPLE (PLEASE DO NOT FILL OUT)

**Place #10:**<br>☐ Home<br>☐ Other:<br>☐ Work<br>☐ School	**Place Name:**<br><br>Full address or two nearest cross streets<br><br>City: Zip:	**Time Arrived:**<br>_________ AM / PM
**How I got here:**<br>☐ Car ☐ Bus/Transit ☐ Bicycle ☐ Walking ☐ Other____		
**What did I do:**<br>☐ Snack ☐ Meal ☐ Beverage ☐ Food Shopping		**Time Left:**<br>__________AM / PM
**Place #11:**<br>☐ Home<br>☐ Other:<br>☐ Work<br>☐ School	**Place Name:**<br><br>Full address or two nearest cross streets<br><br>City: Zip:	**Time Arrived:**<br>_________ AM / PM
**How I got here:**<br>☐ Car ☐ Bus/Transit ☐ Bicycle ☐ Walking ☐ Other__________________		
**What did I do:**<br>☐ Snack ☐ Meal ☐ Beverage ☐ Food Shopping		**Time Left:**<br>__________AM / PM
Time you took the GPS off:_______________12:00 AM / PM<br>**BE SURE TO PLUG IN YOUR GPS TO CHARGE AND PUT RECEIPTS IN ENVELOPE!!!**		
Time(s) removed GPS and reason:8:15-8:30 PM Shower		

DAY 1

Date (m/d/y) ___________

Time you put the GPS on:_______________AM / PM

**Start of Day:**	**Place Name:**		
☐ Home<br>☐ Other:<br>☐ Work<br>☐ School	Full address or two nearest cross streets		
	City:	Zip:	
**What did I do:**<br>☐ Snack ☐ Meal ☐ Beverage ☐ Food Shopping			**Time Left:**<br>________ AM / PM
**Place #1:**<br>☐ Home<br>☐ Other:<br>☐ Work<br>☐ School	**Place Name:**<br>Full address or two nearest cross streets		**Time Arrived:**<br>________ AM / PM
	City:	Zip:	
**How I got here:**<br>☐ Car ☐ Bus/Transit ☐ Bicycle ☐ Walking ☐ Other___________________________________			
**What did I do:**<br>☐ Snack ☐ Meal ☐ Beverage ☐ Food Shopping			**Time Left:**<br>________ AM / PM

**Place #2:**<br>☐ Home<br>☐ Other:<br>☐ Work<br>☐ School	**Place Name:**<br>Full address or two nearest cross streets		**Time Arrived:**<br>_________AM / PM
	City:	Zip:	
**How I got here:**<br>☐ Car ☐ Bus/Transit ☐ Bicycle ☐ Walking ☐ Other____________________________________			
**What did I do:**<br>☐ Snack ☐ Meal ☐ Beverage ☐ Food Shopping			**Time Left:**<br>______ AM / PM
**Place #3:**<br>☐ Home<br>☐ Other:<br>☐ Work<br>☐ School	**Place Name:**<br>Full address or two nearest cross streets		**Time Arrived:**<br>______ AM / PM
	City:	Zip:	
**How I got here:**<br>☐ Car ☐ Bus/Transit ☐ Bicycle ☐ Walking ☐ Other________________			
**What did I do:**<br>☐ Snack ☐ Meal ☐ Beverage ☐ Food Shopping			**Time Left:**<br>_________AM / PM

**Place #4:**<br>☐ Home<br>☐ Other:<br>☐ Work<br>☐ School	**Place Name:**<br><br>Full address or two nearest cross streets<br><br>City: Zip:	**Time Arrived:**<br>__________AM / PM
**How I got here:**<br>☐ Car ☐ Bus/Transit ☐ Bicycle ☐ Walking ☐ Other________________________________		
**What did I do:**<br>☐ Snack ☐ Meal ☐ Beverage ☐ Food Shopping		**Time Left:**<br>__________AM / PM
**Place #5:**<br>☐ Home<br>☐ Other:<br>☐ Work<br>☐ School	**Place Name:**<br><br>Full address or two nearest cross streets<br><br>City: Zip:	**Time Arrived:**<br>__________AM / PM
**How I got here:**<br>☐ Car ☐ Bus/Transit ☐ Bicycle ☐ Walking ☐ Other_______________		
**What did I do:**<br>☐ Snack ☐ Meal ☐ Beverage ☐ Food Shopping		**Time Left:**<br>__________AM / PM

**Place #6:**<br>☐ Home<br>☐ Other:<br>☐ Work<br>☐ School	**Place Name:**<br><br>Full address or two nearest cross streets<br><br>City: Zip:	**Time Arrived:**<br>__________ AM / PM
**How I got here:**<br>☐ Car ☐ Bus/Transit ☐ Bicycle ☐ Walking ☐ Other________________________________		
**What did I do:**<br>☐ Snack ☐ Meal ☐ Beverage ☐ Food Shopping		**Time Left:**<br>__________AM / PM
**Place #7:**<br>☐ Home<br>☐ Other:<br>☐ Work<br>☐ School	**Place Name:**<br><br>Full address or two nearest cross streets<br><br>City: Zip:	**Time Arrived:**<br>__________ AM / PM
**How I got here:**<br>☐ Car ☐ Bus/Transit ☐ Bicycle ☐ Walking ☐ Other_______________		
**What did I do:**<br>☐ Snack ☐ Meal ☐ Beverage ☐ Food Shopping		**Time Left:**<br>__________AM / PM

Place #8: ☐ Home ☐ Other: ☐ Work ☐ School	Place Name:		Time Arrived: __________AM / PM
	Full address or two nearest cross streets		
	City:	Zip:	

How I got here:
☐ Car ☐ Bus/Transit ☐ Bicycle ☐ Walking ☐ Other____

What did I do: ☐ Snack ☐ Meal ☐ Beverage ☐ Food Shopping	Time Left: __________AM / PM

Place #9: ☐ Home ☐ Other: ☐ Work ☐ School	Place Name:		Time Arrived: __________AM / PM
	Full address or two nearest cross streets		
	City:	Zip:	

How I got here:
☐ Car ☐ Bus/Transit ☐ Bicycle ☐ Walking ☐ Other__________________

What did I do: ☐ Snack ☐ Meal ☐ Beverage ☐ Food Shopping	Time Left: __________AM / PM

Place #10: ☐ Home ☐ Other: ☐ Work ☐ School	Place Name:		Time Arrived: __________ AM / PM
	Full address or two nearest cross streets		
	City:	Zip:	

How I got here:
☐ Car ☐ Bus/Transit ☐ Bicycle ☐ Walking ☐ Other____

What did I do: ☐ Snack ☐ Meal ☐ Beverage ☐ Food Shopping	Time Left: _________AM / PM

Place #11: ☐ Home ☐ Other: ☐ Work ☐ School	Place Name:		Time Arrived: ________ AM / PM
	Full address or two nearest cross streets		
	City:	Zip:	

How I got here:
☐ Car ☐ Bus/Transit ☐ Bicycle ☐ Walking ☐ Other__________________

What did I do: ☐ Snack ☐ Meal ☐ Beverage ☐ Food Shopping	Time Left: __________AM / PM

Time you took the GPS off:_______________________AM / PM
BE SURE TO PLUG IN YOUR GPS TO CHARGE AND PUT RECEIPTS IN ENVELOPE!!!

Time(s) removed GPS and reason:

DAY 2

Date (m/d/y) ___________

Time you put the GPS on:_______________AM / PM

**Start of Day:**<br>☐ Home<br>☐ Other:<br>☐ Work<br>☐ School	**Place Name:**<br><br>Full address or two nearest cross streets<br><br>City:	Zip:	
**What did I do:**<br>☐ Snack ☐ Meal ☐ Beverage ☐ Food Shopping			**Time Left:**<br>________ AM / PM
**Place #1:**<br>☐ Home<br>☐ Other:<br>☐ Work<br>☐ School	**Place Name:**<br><br>Full address or two nearest cross streets<br><br>City:	Zip:	**Time Arrived:**<br>________ AM / PM
**How I got here:**<br>☐ Car ☐ Bus/Transit ☐ Bicycle ☐ Walking ☐ Other___________________________________			
**What did I do:**<br>☐ Snack ☐ Meal ☐ Beverage ☐ Food Shopping			**Time Left:**<br>________ AM / PM

**Place #2:**<br>☐ Home<br>☐ Other:<br>☐ Work<br>☐ School	**Place Name:**<br><br>Full address or two nearest cross streets<br><br>City:	Zip:	**Time Arrived:**<br>_________AM / PM
**How I got here:**<br>☐ Car ☐ Bus/Transit ☐ Bicycle ☐ Walking ☐ Other___________________________________			
**What did I do:**<br>☐ Snack ☐ Meal ☐ Beverage ☐ Food Shopping			**Time Left:**<br>______ AM / PM
**Place #3:**<br>☐ Home<br>☐ Other:<br>☐ Work<br>☐ School	**Place Name:**<br><br>Full address or two nearest cross streets<br><br>City:	Zip:	**Time Arrived:**<br>______ AM / PM
**How I got here:**<br>☐ Car ☐ Bus/Transit ☐ Bicycle ☐ Walking ☐ Other________________			
**What did I do:**<br>☐ Snack ☐ Meal ☐ Beverage ☐ Food Shopping			**Time Left:**<br>_________AM / PM

Place #4: ☐ Home ☐ Other: ☐ Work ☐ School	Place Name:		Time Arrived: __________AM / PM
	Full address or two nearest cross streets		
	City:	Zip:	
How I got here: ☐ Car ☐ Bus/Transit ☐ Bicycle ☐ Walking ☐ Other________________________________			
What did I do: ☐ Snack ☐ Meal ☐ Beverage ☐ Food Shopping			Time Left: __________AM / PM
Place #5: ☐ Home ☐ Other: ☐ Work ☐ School	Place Name:		Time Arrived: _________ AM / PM
	Full address or two nearest cross streets		
	City:	Zip:	
How I got here: ☐ Car ☐ Bus/Transit ☐ Bicycle ☐ Walking ☐ Other_________________			
What did I do: ☐ Snack ☐ Meal ☐ Beverage ☐ Food Shopping			Time Left: __________AM / PM

Place #6: ☐ Home ☐ Other: ☐ Work ☐ School	Place Name:		Time Arrived: _________ AM / PM
	Full address or two nearest cross streets		
	City:	Zip:	
How I got here: ☐ Car ☐ Bus/Transit ☐ Bicycle ☐ Walking ☐ Other________________________________			
What did I do: ☐ Snack ☐ Meal ☐ Beverage ☐ Food Shopping			Time Left: _________AM / PM
Place #7: ☐ Home ☐ Other: ☐ Work ☐ School	Place Name:		Time Arrived: _________ AM / PM
	Full address or two nearest cross streets		
	City:	Zip:	
How I got here: ☐ Car ☐ Bus/Transit ☐ Bicycle ☐ Walking ☐ Other_________________			
What did I do: ☐ Snack ☐ Meal ☐ Beverage ☐ Food Shopping			Time Left: __________AM / PM

**Place #8:** ☐ Home ☐ Other: ☐ Work ☐ School	**Place Name:**<br>Full address or two nearest cross streets<br>City: Zip:	**Time Arrived:** __________AM / PM
**How I got here:** ☐ Car ☐ Bus/Transit ☐ Bicycle ☐ Walking ☐ Other____		
**What did I do:** ☐ Snack ☐ Meal ☐ Beverage ☐ Food Shopping		**Time Left:** __________AM / PM

**Place #9:** ☐ Home ☐ Other: ☐ Work ☐ School	**Place Name:**<br>Full address or two nearest cross streets<br>City: Zip:	**Time Arrived:** __________AM / PM
**How I got here:** ☐ Car ☐ Bus/Transit ☐ Bicycle ☐ Walking ☐ Other__________________		
**What did I do:** ☐ Snack ☐ Meal ☐ Beverage ☐ Food Shopping		**Time Left:** __________AM / PM

**Place #10:** ☐ Home ☐ Other: ☐ Work ☐ School	**Place Name:**<br>Full address or two nearest cross streets<br>City: Zip:	**Time Arrived:** __________ AM / PM
**How I got here:** ☐ Car ☐ Bus/Transit ☐ Bicycle ☐ Walking ☐ Other____		
**What did I do:** ☐ Snack ☐ Meal ☐ Beverage ☐ Food Shopping		**Time Left:** _________AM / PM

**Place #11:** ☐ Home ☐ Other: ☐ Work ☐ School	**Place Name:**<br>Full address or two nearest cross streets<br>City: Zip:	**Time Arrived:** _________ AM / PM
**How I got here:** ☐ Car ☐ Bus/Transit ☐ Bicycle ☐ Walking ☐ Other__________________		
**What did I do:** ☐ Snack ☐ Meal ☐ Beverage ☐ Food Shopping		**Time Left:** __________AM / PM

Time you took the GPS off:________________________AM / PM
BE SURE TO PLUG IN YOUR GPS TO CHARGE AND PUT RECEIPTS IN ENVELOPE!!!

Time(s) removed GPS and reason:

DAY 3

Date (m/d/y) ___________

Time you put the GPS on:_______________AM / PM

Start of Day:
☐ Home
☐ Other:
☐ Work
☐ School

Place Name:

Full address or two nearest cross streets

City: Zip:

What did I do:
☐ Snack ☐ Meal ☐ Beverage ☐ Food Shopping

Time Left: ________ AM / PM

Place #1:
☐ Home
☐ Other:
☐ Work
☐ School

Place Name:

Full address or two nearest cross streets

City: Zip:

Time Arrived: ________ AM / PM

How I got here:
☐ Car ☐ Bus/Transit ☐ Bicycle ☐ Walking ☐ Other___________________________________

What did I do:
☐ Snack ☐ Meal ☐ Beverage ☐ Food Shopping

Time Left: ________ AM / PM

Place #2:
☐ Home
☐ Other:
☐ Work
☐ School

Place Name:

Full address or two nearest cross streets

City: Zip:

Time Arrived: _________AM / PM

How I got here:
☐ Car ☐ Bus/Transit ☐ Bicycle ☐ Walking ☐ Other____________________________________

What did I do:
☐ Snack ☐ Meal ☐ Beverage ☐ Food Shopping

Time Left: ______ AM / PM

Place #3:
☐ Home
☐ Other:
☐ Work
☐ School

Place Name:

Full address or two nearest cross streets

City: Zip:

Time Arrived: ______ AM / PM

How I got here:
☐ Car ☐ Bus/Transit ☐ Bicycle ☐ Walking ☐ Other________________

What did I do:
☐ Snack ☐ Meal ☐ Beverage ☐ Food Shopping

Time Left: _________AM / PM

Place #4:
☐ Home
☐ Other:
☐ Work
☐ School

Place Name:

Full address or two nearest cross streets

City: Zip:

Time Arrived:
__________AM / PM

How I got here:
☐ Car ☐ Bus/Transit ☐ Bicycle ☐ Walking ☐ Other____

What did I do:
☐ Snack ☐ Meal ☐ Beverage ☐ Food Shopping

Time Left:
__________AM / PM

Place #5:
☐ Home
☐ Other:
☐ Work
☐ School

Place Name:

Full address or two nearest cross streets

City: Zip:

Time Arrived:
_________ AM / PM

How I got here:
☐ Car ☐ Bus/Transit ☐ Bicycle ☐ Walking ☐ Other__________________

What did I do:
☐ Snack ☐ Meal ☐ Beverage ☐ Food Shopping

Time Left:
__________AM / PM

Place #6:
☐ Home
☐ Other:
☐ Work
☐ School

Place Name:

Full address or two nearest cross streets

City: Zip:

Time Arrived:
__________ AM / PM

How I got here:
☐ Car ☐ Bus/Transit ☐ Bicycle ☐ Walking ☐ Other____

What did I do:
☐ Snack ☐ Meal ☐ Beverage ☐ Food Shopping

Time Left:
__________AM / PM

Place #7:
☐ Home
☐ Other:
☐ Work
☐ School

Place Name:

Full address or two nearest cross streets

City: Zip:

Time Arrived:
_________ AM / PM

How I got here:
☐ Car ☐ Bus/Transit ☐ Bicycle ☐ Walking ☐ Other__________________

What did I do:
☐ Snack ☐ Meal ☐ Beverage ☐ Food Shopping

Time Left:
__________AM / PM

Place #8: ☐ Home ☐ Other: ☐ Work ☐ School	Place Name:	Time Arrived: __________AM / PM
	Full address or two nearest cross streets	
	City: / Zip:	

How I got here:
☐ Car ☐ Bus/Transit ☐ Bicycle ☐ Walking ☐ Other____

What did I do:
☐ Snack ☐ Meal ☐ Beverage ☐ Food Shopping

Time Left: __________AM / PM

Place #9: ☐ Home ☐ Other: ☐ Work ☐ School	Place Name:	Time Arrived: __________AM / PM
	Full address or two nearest cross streets	
	City: / Zip:	

How I got here:
☐ Car ☐ Bus/Transit ☐ Bicycle ☐ Walking ☐ Other__________________

What did I do:
☐ Snack ☐ Meal ☐ Beverage ☐ Food Shopping

Time Left: __________AM / PM

Place #10: ☐ Home ☐ Other: ☐ Work ☐ School	Place Name:	Time Arrived: __________ AM / PM
	Full address or two nearest cross streets	
	City: / Zip:	

How I got here:
☐ Car ☐ Bus/Transit ☐ Bicycle ☐ Walking ☐ Other____

What did I do:
☐ Snack ☐ Meal ☐ Beverage ☐ Food Shopping

Time Left: _________AM / PM

Place #11: ☐ Home ☐ Other: ☐ Work ☐ School	Place Name:	Time Arrived: _________ AM / PM
	Full address or two nearest cross streets	
	City: / Zip:	

How I got here:
☐ Car ☐ Bus/Transit ☐ Bicycle ☐ Walking ☐ Other__________________

What did I do:
☐ Snack ☐ Meal ☐ Beverage ☐ Food Shopping

Time Left: __________AM / PM

Time you took the GPS off:________________________AM / PM
BE SURE TO PLUG IN YOUR GPS TO CHARGE AND PUT RECEIPTS IN ENVELOPE!!!

Time(s) removed GPS and reason:

DAY 4

Date (m/d/y) ___________

Time you put the GPS on:_______________AM / PM

**Start of Day:**<br>☐ Home<br>☐ Other:<br>☐ Work<br>☐ School	**Place Name:**<br>Full address or two nearest cross streets<br>City:	Zip:	
**What did I do:**<br>☐ Snack ☐ Meal ☐ Beverage ☐ Food Shopping			**Time Left:**<br>________ AM / PM
**Place #1:**<br>☐ Home<br>☐ Other:<br>☐ Work<br>☐ School	**Place Name:**<br>Full address or two nearest cross streets<br>City:	Zip:	**Time Arrived:**<br>________ AM / PM
**How I got here:**<br>☐ Car ☐ Bus/Transit ☐ Bicycle ☐ Walking ☐ Other__________________________________			
**What did I do:**<br>☐ Snack ☐ Meal ☐ Beverage ☐ Food Shopping			**Time Left:**<br>________ AM / PM

**Place #2:**<br>☐ Home<br>☐ Other:<br>☐ Work<br>☐ School	**Place Name:**<br>Full address or two nearest cross streets<br>City:	Zip:	**Time Arrived:**<br>_________AM / PM
**How I got here:**<br>☐ Car ☐ Bus/Transit ☐ Bicycle ☐ Walking ☐ Other__________________________________			
**What did I do:**<br>☐ Snack ☐ Meal ☐ Beverage ☐ Food Shopping			**Time Left:**<br>______ AM / PM
**Place #3:**<br>☐ Home<br>☐ Other:<br>☐ Work<br>☐ School	**Place Name:**<br>Full address or two nearest cross streets<br>City:	Zip:	**Time Arrived:**<br>______ AM / PM
**How I got here:**<br>☐ Car ☐ Bus/Transit ☐ Bicycle ☐ Walking ☐ Other_______________			
**What did I do:**<br>☐ Snack ☐ Meal ☐ Beverage ☐ Food Shopping			**Time Left:**<br>_________AM / PM

Place #4:
☐ Home
☐ Other:
☐ Work
☐ School

Place Name:

Full address or two nearest cross streets

City: Zip:

Time Arrived:
__________AM / PM

How I got here:
☐ Car ☐ Bus/Transit ☐ Bicycle ☐ Walking ☐ Other____

What did I do:
☐ Snack ☐ Meal ☐ Beverage ☐ Food Shopping

Time Left:
_______AM / PM

Place #5:
☐ Home
☐ Other:
☐ Work
☐ School

Place Name:

Full address or two nearest cross streets

City: Zip:

Time Arrived:
______ AM / PM

How I got here:
☐ Car ☐ Bus/Transit ☐ Bicycle ☐ Walking ☐ Other__________________

What did I do:
☐ Snack ☐ Meal ☐ Beverage ☐ Food Shopping

Time Left:
__________AM / PM

Place #6:
☐ Home
☐ Other:
☐ Work
☐ School

Place Name:

Full address or two nearest cross streets

City: Zip:

Time Arrived:
_________ AM / PM

How I got here:
☐ Car ☐ Bus/Transit ☐ Bicycle ☐ Walking ☐ Other____

What did I do:
☐ Snack ☐ Meal ☐ Beverage ☐ Food Shopping

Time Left:
_________AM / PM

Place #7:
☐ Home
☐ Other:
☐ Work
☐ School

Place Name:

Full address or two nearest cross streets

City: Zip:

Time Arrived:
_________ AM / PM

How I got here:
☐ Car ☐ Bus/Transit ☐ Bicycle ☐ Walking ☐ Other__________________

What did I do:
☐ Snack ☐ Meal ☐ Beverage ☐ Food Shopping

Time Left:
__________AM / PM

Place #8:
☐ Home
☐ Other:
☐ Work
☐ School

Place Name:

Full address or two nearest cross streets

City: Zip:

Time Arrived:
__________AM / PM

How I got here:
☐ Car ☐ Bus/Transit ☐ Bicycle ☐ Walking ☐ Other______________________________________

What did I do:
☐ Snack ☐ Meal ☐ Beverage ☐ Food Shopping

Time Left:
__________AM / PM

Place #9:
☐ Home
☐ Other:
☐ Work
☐ School

Place Name:

Full address or two nearest cross streets

City: Zip:

Time Arrived:
__________AM / PM

How I got here:
☐ Car ☐ Bus/Transit ☐ Bicycle ☐ Walking ☐ Other_________________

What did I do:
☐ Snack ☐ Meal ☐ Beverage ☐ Food Shopping

Time Left:
__________AM / PM

Place #10:
☐ Home
☐ Other:
☐ Work
☐ School

Place Name:

Full address or two nearest cross streets

City: Zip:

Time Arrived:
_________ AM / PM

How I got here:
☐ Car ☐ Bus/Transit ☐ Bicycle ☐ Walking ☐ Other______________________________________

What did I do:
☐ Snack ☐ Meal ☐ Beverage ☐ Food Shopping

Time Left:
_________AM / PM

Place #11:
☐ Home
☐ Other:
☐ Work
☐ School

Place Name:

Full address or two nearest cross streets

City: Zip:

Time Arrived:
________ AM / PM

How I got here:
☐ Car ☐ Bus/Transit ☐ Bicycle ☐ Walking ☐ Other_________________

What did I do:
☐ Snack ☐ Meal ☐ Beverage ☐ Food Shopping

Time Left:
__________AM / PM

Time you took the GPS off:_______________________AM / PM
BE SURE TO PLUG IN YOUR GPS TO CHARGE AND PUT RECEIPTS IN ENVELOPE!!!

Time(s) removed GPS and reason:

DAY 5

Date (m/d/y) ___________

Time you put the GPS on:_______________AM / PM

**Start of Day:**<br>☐ Home<br>☐ Other:<br>☐ Work<br>☐ School	**Place Name:**<br>Full address or two nearest cross streets<br>City: Zip:	
**What did I do:**<br>☐ Snack ☐ Meal ☐ Beverage ☐ Food Shopping		**Time Left:**<br>________ AM / PM
**Place #1:**<br>☐ Home<br>☐ Other:<br>☐ Work<br>☐ School	**Place Name:**<br>Full address or two nearest cross streets<br>City: Zip:	**Time Arrived:**<br>________ AM / PM
**How I got here:**<br>☐ Car ☐ Bus/Transit ☐ Bicycle ☐ Walking ☐ Other_________________________________		
**What did I do:**<br>☐ Snack ☐ Meal ☐ Beverage ☐ Food Shopping		**Time Left:**<br>________ AM / PM

**Place #2:**<br>☐ Home<br>☐ Other:<br>☐ Work<br>☐ School	**Place Name:**<br>Full address or two nearest cross streets<br>City: Zip:	**Time Arrived:**<br>_________AM / PM
**How I got here:**<br>☐ Car ☐ Bus/Transit ☐ Bicycle ☐ Walking ☐ Other__________________________________		
**What did I do:**<br>☐ Snack ☐ Meal ☐ Beverage ☐ Food Shopping		**Time Left:**<br>______ AM / PM
**Place #3:**<br>☐ Home<br>☐ Other:<br>☐ Work<br>☐ School	**Place Name:**<br>Full address or two nearest cross streets<br>City: Zip:	**Time Arrived:**<br>______ AM / PM
**How I got here:**<br>☐ Car ☐ Bus/Transit ☐ Bicycle ☐ Walking ☐ Other_______________		
**What did I do:**<br>☐ Snack ☐ Meal ☐ Beverage ☐ Food Shopping		**Time Left:**<br>_________AM / PM

Place #4: ☐ Home ☐ Other: ☐ Work ☐ School	Place Name:		Time Arrived: __________AM / PM
	Full address or two nearest cross streets		
	City:	Zip:	
How I got here: ☐ Car ☐ Bus/Transit ☐ Bicycle ☐ Walking ☐ Other____			
What did I do: ☐ Snack ☐ Meal ☐ Beverage ☐ Food Shopping			Time Left: ________AM / PM
Place #5: ☐ Home ☐ Other: ☐ Work ☐ School	Place Name:		Time Arrived: _______ AM / PM
	Full address or two nearest cross streets		
	City:	Zip:	
How I got here: ☐ Car ☐ Bus/Transit ☐ Bicycle ☐ Walking ☐ Other__________________			
What did I do: ☐ Snack ☐ Meal ☐ Beverage ☐ Food Shopping			Time Left: __________AM / PM

Place #6: ☐ Home ☐ Other: ☐ Work ☐ School	Place Name:		Time Arrived: __________ AM / PM
	Full address or two nearest cross streets		
	City:	Zip:	
How I got here: ☐ Car ☐ Bus/Transit ☐ Bicycle ☐ Walking ☐ Other____			
What did I do: ☐ Snack ☐ Meal ☐ Beverage ☐ Food Shopping			Time Left: __________AM / PM
Place #7: ☐ Home ☐ Other: ☐ Work ☐ School	Place Name:		Time Arrived: _________ AM / PM
	Full address or two nearest cross streets		
	City:	Zip:	
How I got here: ☐ Car ☐ Bus/Transit ☐ Bicycle ☐ Walking ☐ Other__________________			
What did I do: ☐ Snack ☐ Meal ☐ Beverage ☐ Food Shopping			Time Left: __________AM / PM

Place #8:
☐ Home
☐ Other:
☐ Work
☐ School

Place Name:

Full address or two nearest cross streets

City: Zip:

Time Arrived:
__________AM / PM

How I got here:
☐ Car ☐ Bus/Transit ☐ Bicycle ☐ Walking ☐ Other______________________________________

What did I do:
☐ Snack ☐ Meal ☐ Beverage ☐ Food Shopping

Time Left:
__________AM / PM

Place #9:
☐ Home
☐ Other:
☐ Work
☐ School

Place Name:

Full address or two nearest cross streets

City: Zip:

Time Arrived:
__________AM / PM

How I got here:
☐ Car ☐ Bus/Transit ☐ Bicycle ☐ Walking ☐ Other_________________

What did I do:
☐ Snack ☐ Meal ☐ Beverage ☐ Food Shopping

Time Left:
__________AM / PM

Place #10:
☐ Home
☐ Other:
☐ Work
☐ School

Place Name:

Full address or two nearest cross streets

City: Zip:

Time Arrived:
_________ AM / PM

How I got here:
☐ Car ☐ Bus/Transit ☐ Bicycle ☐ Walking ☐ Other______________________________________

What did I do:
☐ Snack ☐ Meal ☐ Beverage ☐ Food Shopping

Time Left:
_________AM / PM

Place #11:
☐ Home
☐ Other:
☐ Work
☐ School

Place Name:

Full address or two nearest cross streets

City: Zip:

Time Arrived:
________ AM / PM

How I got here:
☐ Car ☐ Bus/Transit ☐ Bicycle ☐ Walking ☐ Other_________________

What did I do:
☐ Snack ☐ Meal ☐ Beverage ☐ Food Shopping

Time Left:
__________AM / PM

Time you took the GPS off:_______________________AM / PM
BE SURE TO PLUG IN YOUR GPS TO CHARGE AND PUT RECEIPTS IN ENVELOPE!!!

Time(s) removed GPS and reason:

DAY 6

Date (m/d/y) ___________

Time you put the GPS on:_______________AM / PM

**Start of Day:** ☐ Home ☐ Other: ☐ Work ☐ School	**Place Name:**		
	Full address or two nearest cross streets		
	City:	Zip:	
**What did I do:** ☐ Snack ☐ Meal ☐ Beverage ☐ Food Shopping			**Time Left:** ________ AM / PM
**Place #1:** ☐ Home ☐ Other: ☐ Work ☐ School	**Place Name:**		**Time Arrived:** ________ AM / PM
	Full address or two nearest cross streets		
	City:	Zip:	
**How I got here:** ☐ Car ☐ Bus/Transit ☐ Bicycle ☐ Walking ☐ Other___________________________________			
**What did I do:** ☐ Snack ☐ Meal ☐ Beverage ☐ Food Shopping			**Time Left:** ________ AM / PM

**Place #2:** ☐ Home ☐ Other: ☐ Work ☐ School	**Place Name:**		**Time Arrived:** _________AM / PM
	Full address or two nearest cross streets		
	City:	Zip:	
**How I got here:** ☐ Car ☐ Bus/Transit ☐ Bicycle ☐ Walking ☐ Other___________________________________			
**What did I do:** ☐ Snack ☐ Meal ☐ Beverage ☐ Food Shopping			**Time Left:** ______ AM / PM
**Place #3:** ☐ Home ☐ Other: ☐ Work ☐ School	**Place Name:**		**Time Arrived:** ______ AM / PM
	Full address or two nearest cross streets		
	City:	Zip:	
**How I got here:** ☐ Car ☐ Bus/Transit ☐ Bicycle ☐ Walking ☐ Other________________			
**What did I do:** ☐ Snack ☐ Meal ☐ Beverage ☐ Food Shopping			**Time Left:** _________AM / PM

**Place #4:**	**Place Name:**		**Time Arrived:**
☐ Home<br>☐ Other:<br>☐ Work<br>☐ School	Full address or two nearest cross streets		__________AM / PM
	City:	Zip:	
**How I got here:**<br>☐ Car ☐ Bus/Transit ☐ Bicycle ☐ Walking ☐ Other____			
**What did I do:**<br>☐ Snack ☐ Meal ☐ Beverage ☐ Food Shopping			**Time Left:**<br>__________AM / PM
**Place #5:**	**Place Name:**		**Time Arrived:**
☐ Home<br>☐ Other:<br>☐ Work<br>☐ School	Full address or two nearest cross streets		_________ AM / PM
	City:	Zip:	
**How I got here:**<br>☐ Car ☐ Bus/Transit ☐ Bicycle ☐ Walking ☐ Other__________________			
**What did I do:**<br>☐ Snack ☐ Meal ☐ Beverage ☐ Food Shopping			**Time Left:**<br>__________AM / PM

**Place #6:**	**Place Name:**		**Time Arrived:**
☐ Home<br>☐ Other:<br>☐ Work<br>☐ School	Full address or two nearest cross streets		__________ AM / PM
	City:	Zip:	
**How I got here:**<br>☐ Car ☐ Bus/Transit ☐ Bicycle ☐ Walking ☐ Other____			
**What did I do:**<br>☐ Snack ☐ Meal ☐ Beverage ☐ Food Shopping			**Time Left:**<br>__________AM / PM
**Place #7:**	**Place Name:**		**Time Arrived:**
☐ Home<br>☐ Other:<br>☐ Work<br>☐ School	Full address or two nearest cross streets		_________ AM / PM
	City:	Zip:	
**How I got here:**<br>☐ Car ☐ Bus/Transit ☐ Bicycle ☐ Walking ☐ Other__________________			
**What did I do:**<br>☐ Snack ☐ Meal ☐ Beverage ☐ Food Shopping			**Time Left:**<br>__________AM / PM

**Place #8:** ☐ Home ☐ Other: ☐ Work ☐ School	**Place Name:**<br>Full address or two nearest cross streets<br>City: Zip:	**Time Arrived:**<br>__________AM / PM
**How I got here:** ☐ Car ☐ Bus/Transit ☐ Bicycle ☐ Walking ☐ Other______________________________________		
**What did I do:** ☐ Snack ☐ Meal ☐ Beverage ☐ Food Shopping		**Time Left:**<br>__________AM / PM
**Place #9:** ☐ Home ☐ Other: ☐ Work ☐ School	**Place Name:**<br>Full address or two nearest cross streets<br>City: Zip:	**Time Arrived:**<br>__________AM / PM
**How I got here:** ☐ Car ☐ Bus/Transit ☐ Bicycle ☐ Walking ☐ Other_________________		
**What did I do:** ☐ Snack ☐ Meal ☐ Beverage ☐ Food Shopping		**Time Left:**<br>__________AM / PM

**Place #10:** ☐ Home ☐ Other: ☐ Work ☐ School	**Place Name:**<br>Full address or two nearest cross streets<br>City: Zip:	**Time Arrived:**<br>_________ AM / PM
**How I got here:** ☐ Car ☐ Bus/Transit ☐ Bicycle ☐ Walking ☐ Other______________________________________		
**What did I do:** ☐ Snack ☐ Meal ☐ Beverage ☐ Food Shopping		**Time Left:**<br>_________AM / PM
**Place #11:** ☐ Home ☐ Other: ☐ Work ☐ School	**Place Name:**<br>Full address or two nearest cross streets<br>City: Zip:	**Time Arrived:**<br>________ AM / PM
**How I got here:** ☐ Car ☐ Bus/Transit ☐ Bicycle ☐ Walking ☐ Other_________________		
**What did I do:** ☐ Snack ☐ Meal ☐ Beverage ☐ Food Shopping		**Time Left:**<br>__________AM / PM

Time you took the GPS off:________________________AM / PM
BE SURE TO PLUG IN YOUR GPS TO CHARGE AND PUT RECEIPTS IN ENVELOPE!!!

Time(s) removed GPS and reason:

DAY 7

Date (m/d/y) ___________

Time you put the GPS on:_______________AM / PM

**Start of Day:** ☐ Home ☐ Other: ☐ Work ☐ School	**Place Name:**<br>Full address or two nearest cross streets<br>City: Zip:	
**What did I do:** ☐ Snack ☐ Meal ☐ Beverage ☐ Food Shopping		**Time Left:** ________ AM / PM
**Place #1:** ☐ Home ☐ Other: ☐ Work ☐ School	**Place Name:**<br>Full address or two nearest cross streets<br>City: Zip:	**Time Arrived:** ________ AM / PM
**How I got here:** ☐ Car ☐ Bus/Transit ☐ Bicycle ☐ Walking ☐ Other__________________________________		
**What did I do:** ☐ Snack ☐ Meal ☐ Beverage ☐ Food Shopping		**Time Left:** ________ AM / PM

**Place #2:** ☐ Home ☐ Other: ☐ Work ☐ School	**Place Name:**<br>Full address or two nearest cross streets<br>City: Zip:	**Time Arrived:** _________AM / PM
**How I got here:** ☐ Car ☐ Bus/Transit ☐ Bicycle ☐ Walking ☐ Other__________________________________		
**What did I do:** ☐ Snack ☐ Meal ☐ Beverage ☐ Food Shopping		**Time Left:** ______ AM / PM
**Place #3:** ☐ Home ☐ Other: ☐ Work ☐ School	**Place Name:**<br>Full address or two nearest cross streets<br>City: Zip:	**Time Arrived:** ______ AM / PM
**How I got here:** ☐ Car ☐ Bus/Transit ☐ Bicycle ☐ Walking ☐ Other________________		
**What did I do:** ☐ Snack ☐ Meal ☐ Beverage ☐ Food Shopping		**Time Left:** _________AM / PM

Place #4: ☐ Home ☐ Other: ☐ Work ☐ School	Place Name:		Time Arrived: _________AM / PM
	Full address or two nearest cross streets		
	City:	Zip:	
How I got here: ☐ Car ☐ Bus/Transit ☐ Bicycle ☐ Walking ☐ Other________________________________			
What did I do: ☐ Snack ☐ Meal ☐ Beverage ☐ Food Shopping			Time Left: _________AM / PM
Place #5: ☐ Home ☐ Other: ☐ Work ☐ School	Place Name:		Time Arrived: ________ AM / PM
	Full address or two nearest cross streets		
	City:	Zip:	
How I got here: ☐ Car ☐ Bus/Transit ☐ Bicycle ☐ Walking ☐ Other_______________			
What did I do: ☐ Snack ☐ Meal ☐ Beverage ☐ Food Shopping			Time Left: _________AM / PM

Place #6: ☐ Home ☐ Other: ☐ Work ☐ School	Place Name:		Time Arrived: _________ AM / PM
	Full address or two nearest cross streets		
	City:	Zip:	
How I got here: ☐ Car ☐ Bus/Transit ☐ Bicycle ☐ Walking ☐ Other________________________________			
What did I do: ☐ Snack ☐ Meal ☐ Beverage ☐ Food Shopping			Time Left: _________AM / PM
Place #7: ☐ Home ☐ Other: ☐ Work ☐ School	Place Name:		Time Arrived: ________ AM / PM
	Full address or two nearest cross streets		
	City:	Zip:	
How I got here: ☐ Car ☐ Bus/Transit ☐ Bicycle ☐ Walking ☐ Other_______________			
What did I do: ☐ Snack ☐ Meal ☐ Beverage ☐ Food Shopping			Time Left: _________AM / PM

**Place #8:**<br>☐ Home<br>☐ Other:<br>☐ Work<br>☐ School	**Place Name:**<br><br>Full address or two nearest cross streets<br><br>City: Zip:	**Time Arrived:**<br>__________AM / PM
**How I got here:**<br>☐ Car ☐ Bus/Transit ☐ Bicycle ☐ Walking ☐ Other______________________________________		
**What did I do:**<br>☐ Snack ☐ Meal ☐ Beverage ☐ Food Shopping		**Time Left:**<br>__________AM / PM
**Place #9:**<br>☐ Home<br>☐ Other:<br>☐ Work<br>☐ School	**Place Name:**<br><br>Full address or two nearest cross streets<br><br>City: Zip:	**Time Arrived:**<br>__________AM / PM
**How I got here:**<br>☐ Car ☐ Bus/Transit ☐ Bicycle ☐ Walking ☐ Other_________________		
**What did I do:**<br>☐ Snack ☐ Meal ☐ Beverage ☐ Food Shopping		**Time Left:**<br>__________AM / PM

**Place #10:**<br>☐ Home<br>☐ Other:<br>☐ Work<br>☐ School	**Place Name:**<br><br>Full address or two nearest cross streets<br><br>City: Zip:	**Time Arrived:**<br>__________ AM / PM
**How I got here:**<br>☐ Car ☐ Bus/Transit ☐ Bicycle ☐ Walking ☐ Other______________________________________		
**What did I do:**<br>☐ Snack ☐ Meal ☐ Beverage ☐ Food Shopping		**Time Left:**<br>_________AM / PM
**Place #11:**<br>☐ Home<br>☐ Other:<br>☐ Work<br>☐ School	**Place Name:**<br><br>Full address or two nearest cross streets<br><br>City: Zip:	**Time Arrived:**<br>_________ AM / PM
**How I got here:**<br>☐ Car ☐ Bus/Transit ☐ Bicycle ☐ Walking ☐ Other_________________		
**What did I do:**<br>☐ Snack ☐ Meal ☐ Beverage ☐ Food Shopping		**Time Left:**<br>__________AM / PM
Time you took the GPS off:________________________AM / PM<br>**BE SURE TO PLUG IN YOUR GPS TO CHARGE AND PUT RECEIPTS IN ENVELOPE!!!**		
Time(s) removed GPS and reason:		

EXTRA DAY 1

Date (m/d/y) ___________

Time you put the GPS on:_______________AM / PM

**Start of Day:**<br>☐ Home<br>☐ Other:<br>☐ Work<br>☐ School	**Place Name:**<br><br>Full address or two nearest cross streets<br><br>City: Zip:	
**What did I do:**<br>☐ Snack ☐ Meal ☐ Beverage ☐ Food Shopping		**Time Left:**<br>________ AM / PM
**Place #1:**<br>☐ Home<br>☐ Other:<br>☐ Work<br>☐ School	**Place Name:**<br><br>Full address or two nearest cross streets<br><br>City: Zip:	**Time Arrived:**<br>________ AM / PM
**How I got here:**<br>☐ Car ☐ Bus/Transit ☐ Bicycle ☐ Walking ☐ Other___________________________________		
**What did I do:**<br>☐ Snack ☐ Meal ☐ Beverage ☐ Food Shopping		**Time Left:**<br>________ AM / PM

**Place #2:**<br>☐ Home<br>☐ Other:<br>☐ Work<br>☐ School	**Place Name:**<br><br>Full address or two nearest cross streets<br><br>City: Zip:	**Time Arrived:**<br>_________AM / PM
**How I got here:**<br>☐ Car ☐ Bus/Transit ☐ Bicycle ☐ Walking ☐ Other___________________________________		
**What did I do:**<br>☐ Snack ☐ Meal ☐ Beverage ☐ Food Shopping		**Time Left:**<br>_______ AM / PM
**Place #3:**<br>☐ Home<br>☐ Other:<br>☐ Work<br>☐ School	**Place Name:**<br><br>Full address or two nearest cross streets<br><br>City: Zip:	**Time Arrived:**<br>______ AM / PM
**How I got here:**<br>☐ Car ☐ Bus/Transit ☐ Bicycle ☐ Walking ☐ Other________________		
**What did I do:**<br>☐ Snack ☐ Meal ☐ Beverage ☐ Food Shopping		**Time Left:**<br>_________AM / PM

Place #4:
☐ Home
☐ Other:
☐ Work
☐ School

Place Name:

Full address or two nearest cross streets

City: Zip:

Time Arrived:
__________AM / PM

How I got here:
☐ Car ☐ Bus/Transit ☐ Bicycle ☐ Walking ☐ Other______________________________________

What did I do:
☐ Snack ☐ Meal ☐ Beverage ☐ Food Shopping

Time Left:
__________AM / PM

Place #5:
☐ Home
☐ Other:
☐ Work
☐ School

Place Name:

Full address or two nearest cross streets

City: Zip:

Time Arrived:
__________AM / PM

How I got here:
☐ Car ☐ Bus/Transit ☐ Bicycle ☐ Walking ☐ Other_________________

What did I do:
☐ Snack ☐ Meal ☐ Beverage ☐ Food Shopping

Time Left:
__________AM / PM

Place #6:
☐ Home
☐ Other:
☐ Work
☐ School

Place Name:

Full address or two nearest cross streets

City: Zip:

Time Arrived:
_________ AM / PM

How I got here:
☐ Car ☐ Bus/Transit ☐ Bicycle ☐ Walking ☐ Other______________________________________

What did I do:
☐ Snack ☐ Meal ☐ Beverage ☐ Food Shopping

Time Left:
_________AM / PM

Place #7:
☐ Home
☐ Other:
☐ Work
☐ School

Place Name:

Full address or two nearest cross streets

City: Zip:

Time Arrived:
_________ AM / PM

How I got here:
☐ Car ☐ Bus/Transit ☐ Bicycle ☐ Walking ☐ Other_________________

What did I do:
☐ Snack ☐ Meal ☐ Beverage ☐ Food Shopping

Time Left:
__________AM / PM

**Place #8:**	**Place Name:**		**Time Arrived:**
☐ Home<br>☐ Other:<br>☐ Work<br>☐ School	Full address or two nearest cross streets		__________AM / PM
	City:	Zip:	

How I got here:
☐ Car ☐ Bus/Transit ☐ Bicycle ☐ Walking ☐ Other____

**What did I do:**	**Time Left:**
☐ Snack ☐ Meal ☐ Beverage ☐ Food Shopping	__________AM / PM

**Place #9:**	**Place Name:**		**Time Arrived:**
☐ Home<br>☐ Other:<br>☐ Work<br>☐ School	Full address or two nearest cross streets		__________AM / PM
	City:	Zip:	

How I got here:
☐ Car ☐ Bus/Transit ☐ Bicycle ☐ Walking ☐ Other__________________

**What did I do:**	**Time Left:**
☐ Snack ☐ Meal ☐ Beverage ☐ Food Shopping	__________AM / PM

**Place #10:**	**Place Name:**		**Time Arrived:**
☐ Home<br>☐ Other:<br>☐ Work<br>☐ School	Full address or two nearest cross streets		__________ AM / PM
	City:	Zip:	

How I got here:
☐ Car ☐ Bus/Transit ☐ Bicycle ☐ Walking ☐ Other____

**What did I do:**	**Time Left:**
☐ Snack ☐ Meal ☐ Beverage ☐ Food Shopping	_________AM / PM

**Place #11:**	**Place Name:**		**Time Arrived:**
☐ Home<br>☐ Other:<br>☐ Work<br>☐ School	Full address or two nearest cross streets		_________ AM / PM
	City:	Zip:	

How I got here:
☐ Car ☐ Bus/Transit ☐ Bicycle ☐ Walking ☐ Other__________________

**What did I do:**	**Time Left:**
☐ Snack ☐ Meal ☐ Beverage ☐ Food Shopping	__________AM / PM

Time you took the GPS off:________________________AM / PM
BE SURE TO PLUG IN YOUR GPS TO CHARGE AND PUT RECEIPTS IN ENVELOPE!!!

Time(s) removed GPS and reason:

EXTRA DAY 2

Date (m/d/y) ___________

Time you put the GPS on:_______________AM / PM

**Start of Day:**<br>☐ Home<br>☐ Other:<br>☐ Work<br>☐ School	**Place Name:**<br>Full address or two nearest cross streets<br>City: Zip:	
**What did I do:**<br>☐ Snack ☐ Meal ☐ Beverage ☐ Food Shopping		**Time Left:**<br>________ AM / PM
**Place #1:**<br>☐ Home<br>☐ Other:<br>☐ Work<br>☐ School	**Place Name:**<br>Full address or two nearest cross streets<br>City: Zip:	**Time Arrived:**<br>________ AM / PM
**How I got here:**<br>☐ Car ☐ Bus/Transit ☐ Bicycle ☐ Walking ☐ Other___________________________________		
**What did I do:**<br>☐ Snack ☐ Meal ☐ Beverage ☐ Food Shopping		**Time Left:**<br>________ AM / PM

**Place #2:**<br>☐ Home<br>☐ Other:<br>☐ Work<br>☐ School	**Place Name:**<br>Full address or two nearest cross streets<br>City: Zip:	**Time Arrived:**<br>_________AM / PM
**How I got here:**<br>☐ Car ☐ Bus/Transit ☐ Bicycle ☐ Walking ☐ Other___________________________________		
**What did I do:**<br>☐ Snack ☐ Meal ☐ Beverage ☐ Food Shopping		**Time Left:**<br>_______ AM / PM
**Place #3:**<br>☐ Home<br>☐ Other:<br>☐ Work<br>☐ School	**Place Name:**<br>Full address or two nearest cross streets<br>City: Zip:	**Time Arrived:**<br>______ AM / PM
**How I got here:**<br>☐ Car ☐ Bus/Transit ☐ Bicycle ☐ Walking ☐ Other________________		
**What did I do:**<br>☐ Snack ☐ Meal ☐ Beverage ☐ Food Shopping		**Time Left:**<br>_________AM / PM

Place #4: ☐ Home ☐ Other: ☐ Work ☐ School

Place Name:

Full address or two nearest cross streets

City: Zip:

Time Arrived: __________AM / PM

How I got here:
☐ Car ☐ Bus/Transit ☐ Bicycle ☐ Walking ☐ Other____

What did I do:
☐ Snack ☐ Meal ☐ Beverage ☐ Food Shopping

Time Left: __________AM / PM

Place #5: ☐ Home ☐ Other: ☐ Work ☐ School

Place Name:

Full address or two nearest cross streets

City: Zip:

Time Arrived: _________ AM / PM

How I got here:
☐ Car ☐ Bus/Transit ☐ Bicycle ☐ Walking ☐ Other__________________

What did I do:
☐ Snack ☐ Meal ☐ Beverage ☐ Food Shopping

Time Left: __________AM / PM

Place #6: ☐ Home ☐ Other: ☐ Work ☐ School

Place Name:

Full address or two nearest cross streets

City: Zip:

Time Arrived: __________ AM / PM

How I got here:
☐ Car ☐ Bus/Transit ☐ Bicycle ☐ Walking ☐ Other____

What did I do:
☐ Snack ☐ Meal ☐ Beverage ☐ Food Shopping

Time Left: __________AM / PM

Place #7: ☐ Home ☐ Other: ☐ Work ☐ School

Place Name:

Full address or two nearest cross streets

City: Zip:

Time Arrived: _________ AM / PM

How I got here:
☐ Car ☐ Bus/Transit ☐ Bicycle ☐ Walking ☐ Other__________________

What did I do:
☐ Snack ☐ Meal ☐ Beverage ☐ Food Shopping

Time Left: __________AM / PM

Place #8: | **Place Name:** | **Time Arrived:**

☐ Home
☐ Other:
☐ Work
☐ School

Full address or two nearest cross streets

City: | Zip:

__________AM / PM

How I got here:
☐ Car ☐ Bus/Transit ☐ Bicycle ☐ Walking ☐ Other______________________________________

What did I do:
☐ Snack ☐ Meal ☐ Beverage ☐ Food Shopping

Time Left:
__________AM / PM

Place #9: | **Place Name:** | **Time Arrived:**

☐ Home
☐ Other:
☐ Work
☐ School

Full address or two nearest cross streets

City: | Zip:

__________AM / PM

How I got here:
☐ Car ☐ Bus/Transit ☐ Bicycle ☐ Walking ☐ Other_________________

What did I do:
☐ Snack ☐ Meal ☐ Beverage ☐ Food Shopping

Time Left:
__________AM / PM

Place #10: | **Place Name:** | **Time Arrived:**

☐ Home
☐ Other:
☐ Work
☐ School

Full address or two nearest cross streets

City: | Zip:

_________ AM / PM

How I got here:
☐ Car ☐ Bus/Transit ☐ Bicycle ☐ Walking ☐ Other______________________________________

What did I do:
☐ Snack ☐ Meal ☐ Beverage ☐ Food Shopping

Time Left:
_________AM / PM

Place #11: | **Place Name:** | **Time Arrived:**

☐ Home
☐ Other:
☐ Work
☐ School

Full address or two nearest cross streets

City: | Zip:

________ AM / PM

How I got here:
☐ Car ☐ Bus/Transit ☐ Bicycle ☐ Walking ☐ Other_________________

What did I do:
☐ Snack ☐ Meal ☐ Beverage ☐ Food Shopping

Time Left:
__________AM / PM

Time you took the GPS off:_______________________AM / PM
BE SURE TO PLUG IN YOUR GPS TO CHARGE AND PUT RECEIPTS IN ENVELOPE!!!

Time(s) removed GPS and reason:

EXTRA DAY 3

Date (m/d/y) ___________

Time you put the GPS on:_______________AM / PM

Start of Day:
☐ Home
☐ Other:
☐ Work
☐ School

Place Name:

Full address or two nearest cross streets

City:

Zip:

What did I do:
☐ Snack ☐ Meal ☐ Beverage ☐ Food Shopping

Time Left:
________ AM / PM

Place #1:
☐ Home
☐ Other:
☐ Work
☐ School

Place Name:

Full address or two nearest cross streets

City:

Zip:

Time Arrived:
________ AM / PM

How I got here:
☐ Car ☐ Bus/Transit ☐ Bicycle ☐ Walking ☐ Other_________________________________

What did I do:
☐ Snack ☐ Meal ☐ Beverage ☐ Food Shopping

Time Left:
________ AM / PM

Place #2:
☐ Home
☐ Other:
☐ Work
☐ School

Place Name:

Full address or two nearest cross streets

City:

Zip:

Time Arrived:
_________AM / PM

How I got here:
☐ Car ☐ Bus/Transit ☐ Bicycle ☐ Walking ☐ Other_________________________________

What did I do:
☐ Snack ☐ Meal ☐ Beverage ☐ Food Shopping

Time Left:
______ AM / PM

Place #3:
☐ Home
☐ Other:
☐ Work
☐ School

Place Name:

Full address or two nearest cross streets

City:

Zip:

Time Arrived:
______ AM / PM

How I got here:
☐ Car ☐ Bus/Transit ☐ Bicycle ☐ Walking ☐ Other_______________

What did I do:
☐ Snack ☐ Meal ☐ Beverage ☐ Food Shopping

Time Left:
_________AM / PM

Place #4: ☐ Home ☐ Other: ☐ Work ☐ School	Place Name:<br>Full address or two nearest cross streets<br>City: Zip:	Time Arrived: ________AM / PM
How I got here: ☐ Car ☐ Bus/Transit ☐ Bicycle ☐ Walking ☐ Other______________________________		
What did I do: ☐ Snack ☐ Meal ☐ Beverage ☐ Food Shopping		Time Left: ________AM / PM
Place #5: ☐ Home ☐ Other: ☐ Work ☐ School	Place Name:<br>Full address or two nearest cross streets<br>City: Zip:	Time Arrived: _______ AM / PM
How I got here: ☐ Car ☐ Bus/Transit ☐ Bicycle ☐ Walking ☐ Other______________		
What did I do: ☐ Snack ☐ Meal ☐ Beverage ☐ Food Shopping		Time Left: ________AM / PM

Place #6: ☐ Home ☐ Other: ☐ Work ☐ School	Place Name:<br>Full address or two nearest cross streets<br>City: Zip:	Time Arrived: ________ AM / PM
How I got here: ☐ Car ☐ Bus/Transit ☐ Bicycle ☐ Walking ☐ Other______________________________		
What did I do: ☐ Snack ☐ Meal ☐ Beverage ☐ Food Shopping		Time Left: ________AM / PM
Place #7: ☐ Home ☐ Other: ☐ Work ☐ School	Place Name:<br>Full address or two nearest cross streets<br>City: Zip:	Time Arrived: _______ AM / PM
How I got here: ☐ Car ☐ Bus/Transit ☐ Bicycle ☐ Walking ☐ Other______________		
What did I do: ☐ Snack ☐ Meal ☐ Beverage ☐ Food Shopping		Time Left: ________AM / PM

**Place #8:**<br>☐ Home<br>☐ Other:<br>☐ Work<br>☐ School	**Place Name:**<br><br>Full address or two nearest cross streets<br><br>City: Zip:	**Time Arrived:**<br>__________AM / PM
**How I got here:**<br>☐ Car ☐ Bus/Transit ☐ Bicycle ☐ Walking ☐ Other______________________________________		
**What did I do:**<br>☐ Snack ☐ Meal ☐ Beverage ☐ Food Shopping		**Time Left:**<br>__________AM / PM
**Place #9:**<br>☐ Home<br>☐ Other:<br>☐ Work<br>☐ School	**Place Name:**<br><br>Full address or two nearest cross streets<br><br>City: Zip:	**Time Arrived:**<br>__________AM / PM
**How I got here:**<br>☐ Car ☐ Bus/Transit ☐ Bicycle ☐ Walking ☐ Other_________________		
**What did I do:**<br>☐ Snack ☐ Meal ☐ Beverage ☐ Food Shopping		**Time Left:**<br>__________AM / PM

**Place #10:**<br>☐ Home<br>☐ Other:<br>☐ Work<br>☐ School	**Place Name:**<br><br>Full address or two nearest cross streets<br><br>City: Zip:	**Time Arrived:**<br>_________ AM / PM
**How I got here:**<br>☐ Car ☐ Bus/Transit ☐ Bicycle ☐ Walking ☐ Other______________________________________		
**What did I do:**<br>☐ Snack ☐ Meal ☐ Beverage ☐ Food Shopping		**Time Left:**<br>_________AM / PM
**Place #11:**<br>☐ Home<br>☐ Other:<br>☐ Work<br>☐ School	**Place Name:**<br><br>Full address or two nearest cross streets<br><br>City: Zip:	**Time Arrived:**<br>________ AM / PM
**How I got here:**<br>☐ Car ☐ Bus/Transit ☐ Bicycle ☐ Walking ☐ Other_________________		
**What did I do:**<br>☐ Snack ☐ Meal ☐ Beverage ☐ Food Shopping		**Time Left:**<br>__________AM / PM
Time you took the GPS off:_________________________AM / PM<br>**BE SURE TO PLUG IN YOUR GPS TO CHARGE AND PUT RECEIPTS IN ENVELOPE!!!**		
Time(s) removed GPS and reason:		

Office Use Only:

Date Mailed:	Date Returned:

Travel Log Screening & Entry

Initial & Date when completed

Screened	# Valid Days	Follow-Up	Entered	Comments

Participant Follow-Up

Date	Method *(check one)*			Outcome	*Check if follow-up complete*
	Phone	Email	Mail		
